# Safety and Immunogenicity of the Cytomegalovirus Vaccine mRNA-1647 in Healthy Adults: Results from a Phase 2, Randomized, Controlled, Dose-Finding Trial with Long-Term Extension Follow-Up Through Month 48

**DOI:** 10.3390/vaccines14050444

**Published:** 2026-05-16

**Authors:** Carlos Fierro, Daniel Brune, Richard Leggett, James Peterson, Benjamin Lorenz, Renato Calabro Calheiros, Jiang Lin, Anita S. Iyer, Kai Wu, Xin Cao, Alaknanda Kondapally, Sheila Marsh, Shiva Kalidindi, Jennifer Husson, Lori Panther

**Affiliations:** 1 Johnson County Clin-Trials, Lenexa, KS 66219, USA; 2 Optimal Research, Peoria, IL 61614, USA; 3 Crossroads Clinical Research, Victoria, TX 77901, USA; 4 Foothill Family Clinic, Salt Lake City, UT 84109, USA; 5 Moderna, Inc., Cambridge, MA 02142, USAalaknanda.kondapally@modernatx.com (A.K.);; 6 Moderna, Inc., 7550 Wisconsin Ave, Bethesda, MD 20814, USA

**Keywords:** cytomegalovirus, CMV, congenital cytomegalovirus, cCMV, clinical trial, immunogenicity, durability, long-term follow-up, mRNA vaccine, viral vaccine

## Abstract

**Background/Objectives:** No licensed vaccine against cytomegalovirus (CMV) is currently available, despite the significant risk of mother-to-infant transmission leading to serious neurodevelopmental impairment and the substantial morbidity caused by CMV infection in immunocompromised persons. We report results from a phase 2 trial of the investigational CMV mRNA vaccine mRNA-1647 and a long-term extension study (NCT04232280; NCT04975893). **Methods:** This randomized, observer-blind, placebo-controlled phase 2 study, conducted at 9 US sites, enrolled participants in two parts. In the first part, healthy adults aged 18–40 years were stratified by baseline CMV status into CMV-seronegative and CMV-seropositive parallel cohorts and randomized 3:1 to receive mRNA-1647 (50, 100, or 150 μg) or placebo. In the second part, healthy female participants aged 18–40 years were randomized 3:1 to receive 100 μg mRNA-1647 or placebo. In both parts, vaccine or placebo was administered at Months 0, 2, and 6. Participants completing the Primary Trial through Month 18 were eligible to enroll in the extension study, wherein safety and immunogenicity were assessed every 6 months until all participants reached Month 48 (interim analysis) and a subset had Month 54 immunogenicity samples available. Primary objectives were to assess safety and neutralizing antibody responses. **Results:** Solicited adverse reactions were mostly grade 1 or 2 in severity, and no notable dose-related safety trends were identified. Neutralizing antibody and antigen-specific binding IgG responses were induced in CMV-seronegative participants and boosted in CMV-seropositive participants, with durability of responses through Month 48 and up to Month 54. **Conclusions:** The investigational vaccine mRNA-1647 was generally well tolerated and induced durable humoral immune responses across baseline CMV serostatus, with persistence supported through Month 48 and by available Month 54 data.

## 1. Introduction

Cytomegalovirus (CMV) is a widespread β-herpesvirus that can infect people of all ages [1]. After initial (primary) infection, reactivation of latent virus or reinfection with a different CMV strain may occur [1,2]. Most healthy individuals have few signs or symptoms of infection or reinfection [1]. However, transplacental CMV transmission to the fetus during pregnancy can lead to congenital CMV (cCMV) infection, the most common infectious cause of birth defects in the United States, affecting 1 in 200 live births [3,4]. At birth, 10% to 15% of newborns with cCMV infection show symptoms, and about half of these have severe lifelong sequelae, such as sensorineural hearing loss and developmental delay [5,6]. Of the 85% to 90% of newborns with asymptomatic cCMV infection, approximately 10% develop sensorineural hearing loss [5,6,7]. Transmission risk to the fetus is higher from primary infection as compared with nonprimary (i.e., reactivation of latent CMV or reinfection with a different strain) maternal CMV infection during pregnancy [8].

In addition, CMV remains a major cause of morbidity and mortality in immunocompromised populations, particularly hematopoietic stem cell and solid organ transplant recipients, in whom current preventive and therapeutic strategies are limited by toxicity, resistance, and incomplete efficacy, underscoring a substantial unmet need for effective preventive interventions [9].

A safe and effective CMV vaccine is a public health imperative, designated as one of the World Health Organization’s highest priority vaccine targets, especially for women of childbearing age [10,11,12,13,14]. Despite this need, no approved CMV vaccine is currently available, although there have been several candidate vaccines in development [1,11].

The investigational CMV vaccine mRNA-1647 targets CMV gB and the pentamer glycoprotein antigens through the 6 discrete mRNA sequences encapsulated in lipid nanoparticles [15,16]. Both glycoprotein B (gB) and the pentameric gH/gL/UL128/UL130/UL131A glycoprotein complex (pentamer) are essential for CMV infection and facilitate distinct cell entry processes of the virus into multiple cell types [17,18,19,20,21,22]. These antigens have previously been shown to elicit robust neutralizing antibody titers in mice and nonhuman primates [15].

In a phase 1 trial of mRNA-1647 (18−49 years) (NCT03382405), a 3-dose series (30–300 μg) demonstrated an acceptable safety profile and elicited both humoral and cellular immune responses regardless of CMV serostatus [16]. Durable vaccine-induced immunity is critical for a successful CMV vaccine, particularly in females of childbearing age and high-risk individuals, as the required duration of protection may be variable and, in some cases, prolonged [23,24,25]. Currently, the durability of mRNA-1647-induced immune responses beyond Month 18 remains to be established.

The primary objectives of the randomized, observer-blind, placebo-controlled, dose-finding phase 2 trial (Primary Trial) were to evaluate the safety and antibody-mediated immune responses of mRNA-1647 across 3 dose levels in healthy adults aged 18 to 40 years to inform dose-level selection for further development. The primary objectives of the long-term extension of the phase 2 trial (Extension Trial) were to evaluate longer-term antibody-mediated immune persistence of mRNA-1647.

## 2. Methods

### 2.1. Trial Design and Participants

The Primary Trial (phase 2) was conducted in healthy CMV-seropositive and CMV-seronegative participants aged 18 to 40 years at 9 US sites between 9 January 2020 and 4 January 2023, in two parts (NCT04232280; Figure S1; full inclusion and exclusion criteria in Supplemental Materials). A CMV IgG assay based on recombinant CMV antigens not encoded by mRNA-1647 (Supplementary Material) was used to determine CMV serostatus at screening and monitor for primary CMV infection during the trial. In Part 1, participants were randomly assigned 3:1 to receive mRNA-1647 (50 μg, 100 μg, or 150 μg) or a placebo. In Part 2, female participants were randomly assigned 3:1 to receive mRNA-1647 100 μg or a placebo. In both parts, mRNA-1647 or placebo was administered in a 3-dose series following a 0-, 2-, 6-month schedule on days 1, 56, and 168. The trial duration for each participant was 18 months, with the trial lasting a total of 3 years.

CMV serostatus was determined at screening using a CMV IgG assay based on recombinant CMV antigens not encoded by mRNA-1647 (Supplementary Material).

The phase 2 Extension Trial (NCT04975893) was conducted in a subset of healthy adult participants aged 18–40 years who completed the randomized Primary Trial. Participants of the Primary Trial were eligible for the Extension Trial if they were randomly assigned to receive mRNA-1647 (50 μg, 100 μg, or 150 μg; CMV-seronegative or CMV-seropositive) or placebo (CMV-seronegative) in the Primary Trial. CMV-seronegative participants were only included if they had not seroconverted before enrollment in the Extension Trial (full eligibility criteria in Supplementary Materials). Extension Trial results are based on interim analysis at Month 48 (data cutoff of 9 May 2025), when many participants had already been evaluated for safety and immunogenicity out to Month 54.

### 2.2. Randomization, Blinding, and Ethics

Randomization was stratified by CMV serostatus via an interactive response technology in a sequential manner in study Parts 1 and 2. The trial was observer-blind and only unblinded to designated personnel who prepared/administered the injection. A blinded internal safety team monitored participant safety, with ad hoc safety reviews by an unblinded independent safety monitoring committee.

**Figure 1:**
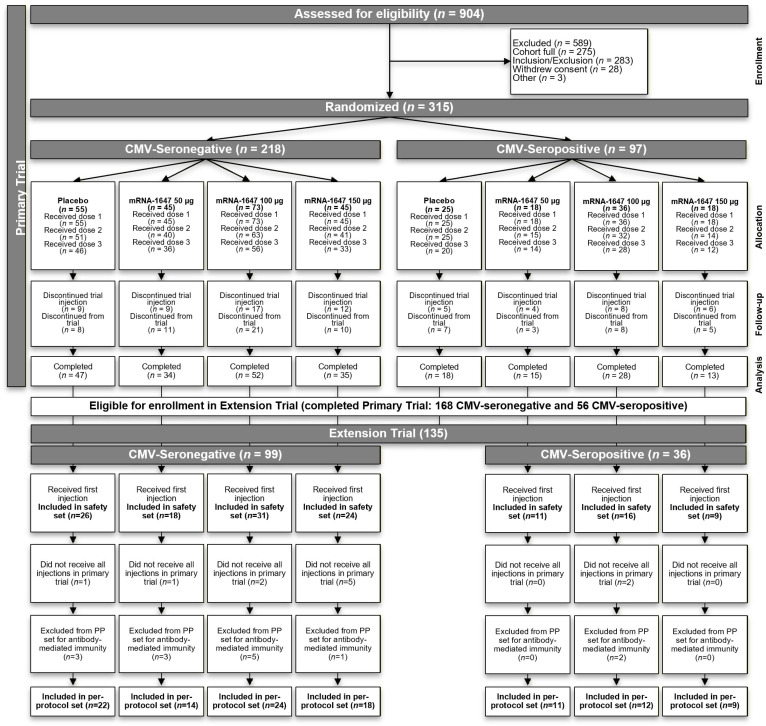
Participant disposition by CMV serostatus in the Primary and Extension Trials. The flow of CMV-seronegative and CMV-seropositive participants by vaccination group and dose. In the randomized set (Primary Trial), the most frequent reasons for discontinuation of trial injection were consent withdrawal (*n* = 32, 10.2%), lost to follow-up (*n* = 14, 4.4%), and AEs (*n* = 13, 4.1%), and the most common reasons for discontinuation from the trial were consent withdrawal (*n* = 42, 13.3%) and lost to follow-up (*n* = 27, 8.6%). Other reasons for discontinuation included physician decision, pregnancy, protocol deviation, or other unspecified reason.

The trial protocol, amendments, and informed consent form were reviewed and approved by the Advarra Institutional Review Board. Participants provided written informed consent before trial entry and procedures. Both the Primary and Extension Trials were conducted according to their protocols; applicable national, state, and local laws or regulations; and the principles of the International Conference on Harmonisation Tripartite Guideline E6(R2): Good Clinical Practice and the Declaration of Helsinki. This manuscript was prepared in accordance with the CONSORT 2025 guidelines for randomized clinical trials, and the CONSORT flow diagram (Figure 1) and checklist have been provided.

### 2.3. Trial Objectives and Endpoints

The primary objectives were to evaluate safety and the nAb responses against epithelial cell and fibroblast infection following vaccination with mRNA-1647 at different dose levels administered on a 3-dose (0-, 2-, 6-month) schedule. The secondary objectives were to evaluate antigen-specific binding IgG responses and to evaluate immune responses by CMV serostatus following vaccination with mRNA-1647 at different dose levels administered on a 3-dose (0-, 2-, 6-month) schedule.

The primary objective of the Extension Trial was to evaluate the long-term antibody-mediated immunogenicity from the final visit in the Primary Trial (Month 18) through Month 54. The secondary objective was to evaluate the long-term safety of mRNA-1647 through Month 54; assessments include adverse events (AEs) leading to discontinuation from the trial, serious AEs (SAEs), and deaths.

### 2.4. Vaccine

mRNA-1647 is an investigational mRNA-based CMV vaccine that consists of 6 distinct mRNA sequences encoding 2 CMV glycoprotein antigens encapsulated in a lipid nanoparticle formulation. One mRNA sequence encodes the full-length CMV gB antigen, and 5 mRNA sequences encode the glycoprotein subunits of the CMV pentamer antigen. mRNA-1647 was lyophilized and reconstituted for administration. Placebo consisted of a 0.9% sodium chloride injection (USP; normal saline). Injections were administered intramuscularly into the deltoid muscle.

### 2.5. Safety

Safety assessments included solicited local and systemic ARs collected through 7 days and unsolicited AEs collected through 28 days after each dose. Medically attended adverse events (MAAEs) and serious AEs were collected through 6 months after the last dose and throughout the Primary Trial, respectively. In the Extension Trial, long-term safety of mRNA-1647 was evaluated through Month 54, including assessment of AEs leading to discontinuation from the trial, SAEs, and deaths.

### 2.6. Immunogenicity

Blood samples for assessing antibody-mediated immunity were collected on days 1, 29, 56, 84, 168, 196, 336, and 504 (Months 0, 1, 2, 3, 6, 7, 12, and 18) in the Primary Trial and at Months 24, 30, 36, 42, 48, and 54 in the Extension Trial.

Microneutralization (MN) and enzyme-linked immunosorbent assay (ELISA) methods were both performed at Cerba Research (formerly Viroclinics, Rotterdam, The Netherlands) using fit-for-purpose validated assays to quantify neutralizing (nAb) and binding (bAb) antibody titers respectively.

***Neutralizing antibody (nAb) assay*****.** Briefly, in-house stocks of human cytomegalovirus (hCMV) strains AD169 and VR1814 were independently incubated with serial dilutions of heat-inactivated serum samples. The resulting virus–serum mixtures were applied to confluent monolayers of HEL-299 fibroblasts (for AD169) or ARPE-19 epithelial cells (for VR1814). For the fibroblast-based MN assay, 5% guinea pig complement was included in the virus-serum mixture.

Following incubation for 17–20 h at 37 °C in a humidified incubator with 5% CO_2_, viral infection was quantified using an indirect enzyme-based detection method. This involved a primary mouse monoclonal antibody targeting hCMV immediate early (IE1) protein (clone 8.B1.2; Millipore, Amsterdam, The Netherlands, Cat# MAB810), followed by detection of this antibody by the addition of horseradish peroxidase (HRP)-conjugated goat anti-mouse IgG secondary antibody (Life Technologies, Bleiswijk, The Netherlands, Cat# A16072). Viral infection was then measured by development with TrueBlue substrate and viral spots were counted using SX Ultimate-V Analyzer (series 5 or 6), Cellular Technology Limited (CTL) or equivalent.

Neutralizing antibody titers were defined as the reciprocal of serum dilution resulting in 50% inhibition of viral spot counts relative to serum-free, virus-only control wells.

***Binding antibody (bAb) assay.*** Serially diluted serum samples, including positive controls, were added to 96-well plates coated with hCMV glycoprotein B (gB) (Sino Biological, Eschborn, Germany, Cat# 10202-V08HI) or pentamer antigen (Native Antigen Company, Kidlington, United Kingdom, Cat# CMV-pent).

Following incubation, bound antibodies were detected using a horseradish peroxidase (HRP)-conjugated goat anti-human IgG secondary antibody (Thermo Scientific, Bleiswijk, The Netherlands, Cat# 31410) and visualized with 3,3′,5,5′-tetramethylbenzidine (TMB) substrate (Sigma, Amsterdam, The Netherlands, Cat# TO440-100 mL). Optical density (OD) was measured at 450 nm with a reference wavelength of 620 nm (OD_450–620_).

Binding antibody endpoint titers were determined using a four-parameter logistic (4PL) curve by interpolating sample OD values against the standard positive control included on each plate. Plate acceptance criteria were defined by mean IC_50_ values of 3.57 ± 0.42 log_10_ for the gB ELISA and 2.99 ± 0.80 log_10_ for the pentamer ELISA, based on the positive control serum.

### 2.7. Statistics

Sample size was sufficient to descriptively summarize the long-term safety and immunogenicity of mRNA-1647 without formal hypothesis testing. Analyses were performed using SAS^®^ Version 9.4 or higher (SAS Institute, Cary, NC, USA). Data from Parts 1 and 2 were combined and presented by CMV serostatus and vaccination group. Analysis sets are defined in Supplementary Materials.

Continuous variables and categorical variables were summarized using descriptive statistics. Baseline value, unless specified otherwise, was defined as the most recent non-missing measurement collected before dose 1. For immunogenicity outcomes, GMTs with 95% confidence intervals (CIs) were provided by serostatus for each vaccination group and timepoint; 95% CIs were based on the t-distribution of log-transformed values that were back-transformed to the original scale.

## 3. Results

### 3.1. Trial Participants

In the Primary Trial, a total of 315 participants were randomized (Figure 1) in two parts. In Part 1, 252 participants of both sexes were randomized 3:1 to receive mRNA-1647 (50 μg, 100 μg, or 150 μg) or placebo. In Part 2, 63 female participants were randomized 3:1 to receive mRNA-1647 100 μg or placebo. In both Parts, CMV-seronegative and CMV-seropositive participants were enrolled, and vaccine or placebo was administered in a 3-dose series (Months 0, 2, and 6; administered on days 1, 56, and 168; Figure S1). The results from Parts 1 and 2 are reported together.

Of the 315 participants at baseline, 215 were CMV-seronegative and 100 were CMV-seropositive (Table 1). These baseline counts differ slightly from screening serostatus (218 CMV-seronegative, 97 CMV-seropositive) because baseline CMV serology was considered positive for participants who were seronegative at screening if their neutralizing antibody titers against epithelial-cell or fibroblast infection were above the lower limit of quantification at baseline (2 in the placebo group and 1 in the 100 µg mRNA-1647 treatment group). The mean (standard deviation) age was 28.6 (6.7) and 31.2 (6.5) years in CMV-seronegative and CMV-seropositive participants, respectively. Overall, 65.4% were female (due to enrollment of only females in Part 2), 84.1% were White, and 17.5% were Hispanic or Latino. All enrolled participants (*n* = 315) received ≥1 dose of mRNA-1647 or placebo, with 276 (87.6%) receiving 2 doses and 245 (77.8%) receiving 3 doses. A total of 242 participants (76.8%) completed the Primary Trial through 18 months per participant.

Participants completing the Primary Trial were eligible for continued follow-up through enrollment in the Extension Trial (Figure 1). A total of 135 participants enrolled: 99 CMV-seronegative and 36 CMV-seropositive. CMV-seronegative participants were only enrolled in the Extension Trial if they remained CMV-seronegative (serum IgG against CMV antigens not encoded by mRNA-1647; see Supplementary Materials). Full inclusion/exclusion criteria are in the Supplementary Material.

Of the 135 Extension Trial participants, 91.9% (124) had received all 3 study injections in the Primary Trial (Figure 1) and 81.5% (110) met the per-protocol set criteria for antibody-mediated immunogenicity (defined in Supplementary Materials). Demographic characteristics were generally well matched across treatment and CMV serostatus groups (Table S1). The long-term follow-up data are presented for antibody-mediated immunity through Month 54, based on an interim analysis performed when all participants had reached Month 48; at that time, Month 54 samples were available for 56% of participants in the per-protocol set. Accordingly, Month 54 results should be interpreted as based on a subset of participants.

### 3.2. Safety

#### 3.2.1. Solicited Adverse Reactions

**Figure 2:**
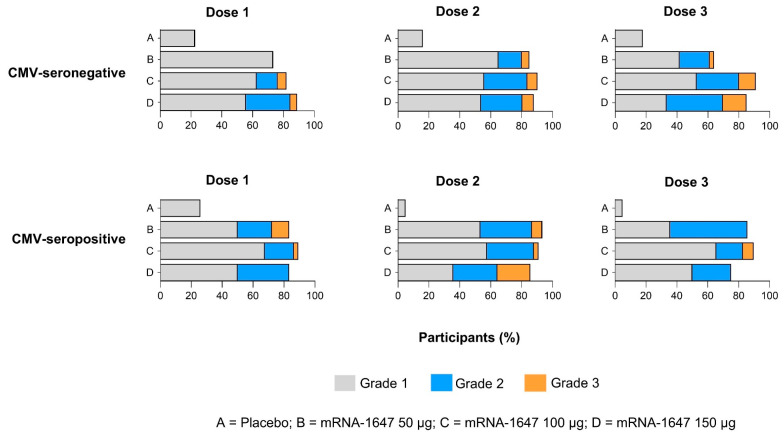
Any solicited local adverse reaction by CMV serostatus. Solicited local adverse reactions in CMV-seronegative and CMV-seropositive participants by vaccination group and grade following each dose. Data are from doses 1 (CMV-seronegative, *n* = 215; CMV-seropositive, *n* = 100), 2 (CMV-seronegative, *n* = 192; CMV-seropositive, *n* = 83), and 3 (CMV-seronegative, *n* = 169; CMV-seropositive, *n* = 76) solicited safety sets. As depicted in the key, values for grades 1 (grey bars), 2 (blue bars), and 3 (orange bars) are shown for placebo (A rows), mRNA-1647 50 μg (B rows), mRNA-1647 100 μg (C rows), and mRNA-1647 150 μg (D rows). CMV, cytomegalovirus.

The incidence of any local solicited adverse reaction (AR) was similar between the CMV-seronegative and CMV-seropositive groups following doses 1, 2, or 3 in the pooled-dose mRNA-1647 groups (50 μg, 100 μg, and 150 μg). The incidence of any local ARs was higher with mRNA-1647 than with placebo (Figure 2). Most local solicited ARs were grade 1 or 2 in severity, and no grade 4 reactions were reported. No clear dose-related trends were observed in the incidence or severity of local ARs after doses 1, 2, or 3. Injection-site pain was the most frequently reported local AR in both the pooled-dose mRNA-1647 and placebo groups after each dose (Figures S2 and S3). Individually, local ARs occurred with higher incidence in mRNA-1647 recipients than in placebo recipients, with no notable trends by dose or CMV serostatus (Figures S2 and S3).

**Figure 3:**
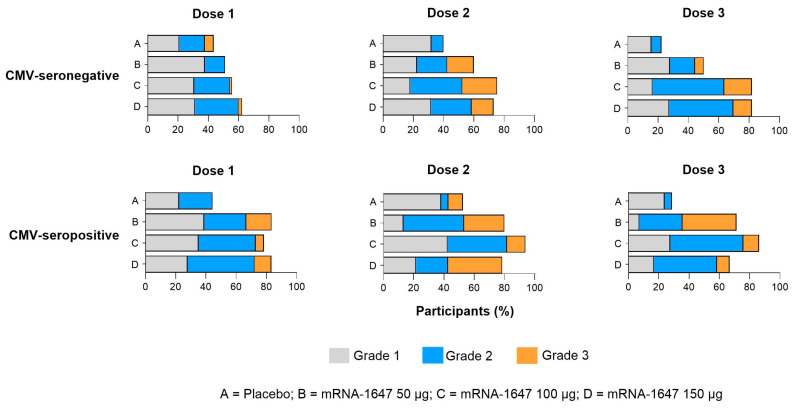
Any solicited systemic adverse reaction by CMV serostatus. Solicited systemic adverse reactions in CMV-seronegative and CMV-seropositive participants by vaccination group and grade following each dose. Data are from doses 1 (CMV-seronegative, *n* = 215; CMV-seropositive, *n* = 100), 2 (CMV-seronegative, *n* = 192; CMV-seropositive, *n* = 83), and 3 (CMV-seronegative, *n* = 169; CMV-seropositive, *n* = 76) solicited safety sets. As depicted in the key, values for grades 1 (grey bars), 2 (blue bars), and 3 (orange bars) are shown for placebo (A rows), mRNA-1647 50 μg (B rows), mRNA-1647 100 μg (C rows), and mRNA-1647 150 μg (D rows). CMV, cytomegalovirus.

Systemic solicited ARs showed no clear dose-related trends in severity. In the pooled-dose mRNA-1647 group, the proportion of participants reporting any systemic solicited AR was higher than in the placebo group after doses 1, 2, and 3 (Figure 3). Most systemic ARs were grade 1 or 2 in severity. No notable trends were observed related to the dose of mRNA-1647 in the incidence or severity of systemic ARs after doses 1, 2, or 3. However, the mRNA-1647 pooled-dose groups showed a higher incidence of any systemic ARs in CMV-seropositive than CMV-seronegative participants after dose 1; these differences decreased after doses 2 and 3. Headache, fatigue, and myalgia were the most commonly reported individual systemic ARs after mRNA-1647 vaccination across serostatus (Figures S4 and S5).

#### 3.2.2. Unsolicited Adverse Events

In the Primary Trial, the incidences of any unsolicited adverse events (AEs), irrespective of their assessed relationship to trial injection, were similar across serostatus subgroups in the pooled-dose mRNA-1647 and placebo groups (Table S2). Serious AEs occurred in 2 participants in the mRNA-1647 100-μg group (1 CMV-seronegative [anxiety and depression] and 1 CMV-seropositive [follicular thyroid cancer]) and were deemed unrelated to the study vaccine. AEs leading to discontinuation of trial vaccination, regardless of relationship, were reported by 1.9% and 2.7% of CMV-seronegative and CMV-seropositive participants, respectively, in the pooled-dose mRNA-1647 group, compared with 0% and 3.7% in the placebo group. AEs considered related to study vaccines by the investigator occurred in 0.6% and 1.4% of CMV-seronegative and CMV-seropositive participants, respectively, and none occurred in the placebo group. Severe AEs in >1 participant following mRNA-1647 vaccination were decreased hemoglobin level, prolonged activated partial thromboplastin time, prolonged prothrombin time, influenza, depression, migraine, and elevated blood pressure. Among placebo recipients, severe AEs in >1 participant were decreased hemoglobin level and prolonged activated partial thromboplastin time. The most common reported MAAEs in the pooled-dose mRNA-1647 group included infections and infestations, general disorders and injection site conditions, skin and subcutaneous tissue disorders, nervous system disorders, psychiatric disorders, and investigations (including alanine aminotransferase increase and hemoglobin decrease). No deaths were reported and there were no AEs leading to trial discontinuation during the Primary Trial.

In the Extension Trial from Months 18 through at least Month 48, 7 serious adverse events (SAEs) occurred in 6/135 participants (4.4%); of these, 6 were reported in 5/99 (5.1%) CMV-seronegative participants and 1 was reported in 1/36 (2.8%) CMV-seropositive participants (Table S3). None of the SAEs were considered by the investigator as related to the trial injections received during the Primary Trial. The SAEs in CMV-seronegative participants included 2 seizures (1 participant, 150 μg), 2 spontaneous abortions (1 each in 100 μg and placebo), 1 trigeminal neuralgia (100 μg), and 1 upper limb fracture (100 μg). The SAE in the CMV-seropositive participant (100 μg) was a urinary tract infection. Seven pregnancies were reported in 5 participants through at least Month 48; pregnancy outcomes were term birth without complications (*n* = 2, placebo), normal delivery (*n* = 1, mRNA-1647 100 μg), preterm birth (*n* = 1, placebo), and spontaneous abortion (*n* = 1, mRNA-1647 100 μg; *n* = 2, placebo). No deaths and no AEs leading to trial discontinuation were reported through at least Month 48 of the Extension Trial.

### 3.3. Immunogenicity

The baseline geometric mean titer (GMT) of all CMV-seropositive participants in the Primary Trial was used as the benchmark level of natural infection, against which post-vaccination neutralizing (nAb) and binding (bAb) antibody responses were compared.

#### 3.3.1. Neutralizing Antibodies

**Figure 4:**
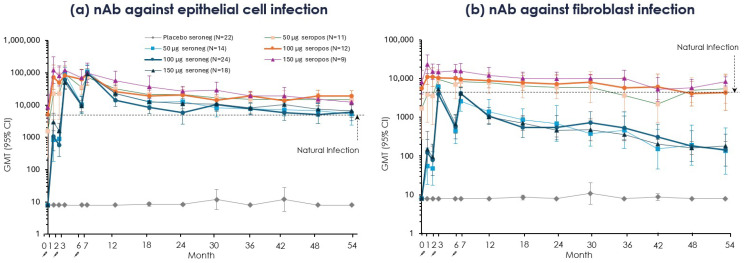
Neutralizing antibody titers against CMV infection through Month 54 by CMV serostatus. Data are represented by serostatus, vaccination group, and visit as GMT and corresponding 95% CI. (**a**) nAb titers against epithelial cell infection and (**b**) nAb titers against fibroblast infection are presented from day 1 (Month 0) through Month 54. Primary Trial time points are Months 0–18; extension follow-up time points are Months 24, 30, 36, 42, 48, and 54. CMV-seronegative (red hues) and CMV-seropositive (blue hues) vaccination groups were plotted over time. Doses 1, 2, and 3 were administered on day 1, Month 2, and Month 6, respectively, as represented by an arrow and syringe. The horizontal dashed line indicates the baseline GMT of all CMV-seropositive participants (i.e., naturally infected benchmark; epithelial cells 4575.7 and fibroblasts 4215.5). Data are from the per-protocol set; n may vary by time point. Note that larger CIs, such as those visible in the placebo, are mostly due to outliers and the log scale. CMV, cytomegalovirus; D, day; GMT, geometric mean titer; M, month; nAb, neutralizing antibody.

*In CMV-seronegative groups*, nAb GMTs against *epithelial cell* infection increased after each mRNA-1647 dose and stayed above the seropositive benchmark, or overlapped with it, throughout long-term follow-up up to Month 54 (Figure 4a, Tables S4 and S5), consistent with a durable response. On the contrary, *fibroblast* nAb GMTs at peak level (at one month post doses 2 or 3) approached the level of the seropositive benchmark and then fell below it, declining more steeply at first and then more gradually, although remaining consistently above baseline and the seronegative placebo level through Month 54 (Figure 4b).

In *CMV-seropositive groups*, nAb GMTs against *epithelial cell* infection increased after the first dose and then stayed above the baseline benchmark throughout Month 54, consistent with maintenance of boosted responses over time (Figure 4a). A similar pattern of nAb GMTs against *fibroblast* infection was observed, although they were closer to the benchmark and overlapped with it.

#### 3.3.2. Binding Antibodies

The mRNA-1647-induced anti-pentamer and anti-gB IgG GMTs were also assessed against the benchmark level of natural infection (the baseline GMT of all CMV-seropositive participants).

**Figure 5:**
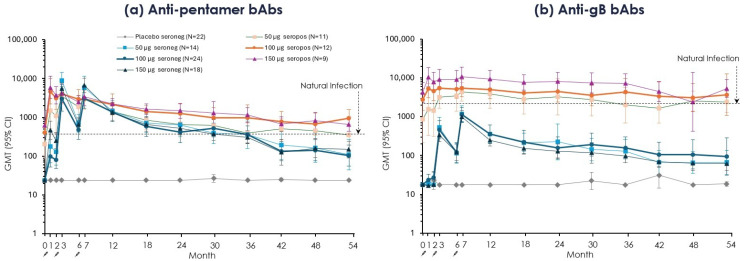
Binding IgG titers against mRNA-1647 antigens through Month 54 by CMV serostatus. Data are represented by serostatus, vaccination group, and visit as GMT and corresponding 95% CI. (**a**) anti-pentamer binding IgG titers and (**b**) anti-gB binding IgG titers are presented from day 1 (Month 0) through Month 54. Primary Trial time points are Months 0–18; extension follow-up time points are Months 24, 30, 36, 42, 48, and 54. CMV-seronegative (red hues) and CMV-seropositive (blue hues) vaccination groups were plotted over time. Doses 1, 2, and 3 were administered on day 1, Month 2, and Month 6, respectively, as represented by an arrow and syringe. The horizontal dashed line indicates the baseline GMT of all CMV-seropositive participants (i.e., naturally infected benchmark; anti-pentamer 400.2 and anti-gB 2400.9). Data are from the per-protocol set; n may vary by time point. bAb, binding antibody; CMV, cytomegalovirus; D, day; gB, glycoprotein B; GMT, geometric mean titer; IgG, immunoglobulin G; M, month.

*Anti-pentamer* IgG GMTs in *CMV-seronegative* groups peaked at one month after the second and third doses when they substantially exceeded the seropositive benchmark (Figure 5a). Subsequently, anti-pentamer GMTs waned to approximately the seropositive benchmark level between Months 24 and 36 and below it from Months 42 to 54 (Figure 5a), although staying substantially above the seronegative placebo level throughout. In *CMV-seropositive* groups, *anti-pentamer* GMTs generally exceeded the seropositive benchmark after all doses through Month 54 (Figure 5a).

*Anti-gB* responses demonstrated a pattern similar to that of anti-pentamer responses in both serostatus groups. However, unlike the anti-pentamer GMTs described above, anti-gB GMTs in seronegative participants did not exceed the benchmark GMT at any timepoint although they remained above placebo GMTs (Figure 5b). In *seropositive participants*, anti-gB GMTs, as with anti-pentamer GMTs, peaked one month after the first injection and did not increase further after the second or third injections. From their peak levels, anti-gB GMTs induced in seropositive participants by the highest mRNA-1647 doses (100 and 150 μg) typically exceeded the seropositive benchmark level until Month 42 (Figure 5b).

## 4. Discussion

The public health rationale for a safe, effective, and durable CMV vaccine remains compelling. Global CMV seroprevalence is estimated at 83% overall and 86% in women of reproductive age [26], and multiple public health agencies have prioritized CMV prevention and vaccine development [11,12,13,14].

Across this phase 2 randomized Primary Trial and through to the Extension Trial, mRNA-1647 showed an acceptable safety profile across dose levels (50 μg, 100 μg, and 150 μg) regardless of CMV serostatus. The neutralizing and binding antibody responses did not show a clear dose-related pattern over follow-up. Antigen-specific binding and neutralizing antibody levels generally peaked about 1 month after the third injection and then waned or stabilized, as described for other vaccine platforms [27,28,29,30,31].

The Primary Trial data did not demonstrate dose-related trends in solicited adverse reactions (ARs) or unsolicited adverse events (AEs). As expected for an immunogenic vaccine, solicited ARs occurred more frequently with mRNA-1647 than with placebo after each dose in both serostatus groups. CMV-seropositive recipients reported more systemic ARs than CMV-seronegative recipients after dose 1; that difference diminished after dose 2 and disappeared after dose 3. This pattern may reflect vaccine-induced immune activation in the setting of preexisting immunity or prior vaccination and is generally consistent with published data for mRNA and non-mRNA vaccines [32,33,34,35].

mRNA-1647-induced nAb against epithelial cell infection was above or similar to the naturally infected benchmark through Month 54 in both serostatus groups, while pentamer-binding IgG exceeded or equaled the benchmark level through Month 54 for the CMV-seropositive group and through Month 36 for the CMV-seronegative group. In contrast, both gB-binding IgG and nAb against fibroblast infection at their peaks in *CMV-seronegative participants* were only at or near the level of the natural immunity benchmark and then declined gradually, although they remained above placebo out to Month 54. In *CMV-seropositive participants*, gB-binding IgG and fibroblast-infection nAb were boosted, declining gradually to the natural infection level by about Month 36. These antibody data are consistent with those from the phase 1 trial of mRNA-1647 [16,36].

The observation that mRNA-1647-induced pentamer-binding IgG titers and epithelial-cell neutralizing titers were generally higher than natural infection levels, whereas gB-binding IgG titers and fibroblast neutralizing titers were generally lower than natural infection levels, may reflect the distinct CMV entry complexes required for epithelial versus fibroblast infection. Epithelial-cell infection depends primarily on the viral pentamer (pentameric gH/gL/UL128/UL130/UL131A glycoprotein complex) [37,38], while fibroblast infection relies primarily on the viral trimer complex (gH/gL/gO) [39,40], although entry into both cell types ultimately requires gB-mediated fusion [41,42].

mRNA-1647 has been compared with other experimental CMV vaccines. Supporting the durability of the immune response induced by mRNA-1647, the vaccine-induced nAb appeared to wane more slowly than those of the replication-defective CMV candidate V160 [43]. Additionally, the humoral response to mRNA-1647 in its first-in-human study has been compared with the experimental CMV vaccine gB/MF59 (phase 2 study): mRNA-1647 showed lower gB-specific IgG and antibody-dependent cellular phagocytosis responses but higher nAb and antibody-dependent cellular cytotoxicity responses [36].

Although we did not directly assess memory B cells in this study, data from the phase 1 study showed that mRNA-1647 induces durable gB- and pentamer-specific IgG-secreting memory B-cell responses for at least 12 months [44], and the sustained antibody responses in this study likely reflect persistence of antigen-specific memory B-cell pools. Similarly, antigen-specific T-cell responses were also assessed in the phase 1 study, and mRNA-1647 elicited robust and polyfunctional (≥2 functional markers) Th1-biased CD4+ and CD8+ T-cell responses [44].

The phase 2 data reported herein, and interpreted alongside the phase 1 results [16], supported the selection of the 100-μg dose for the phase 3 trial in participants 16 to 40 years of age (NCT05085366). The major findings from these phase 2 studies are that mRNA-1647 induced immune responses that persisted for at least 4.5 years, providing evidence that mRNA-based vaccines can elicit long-term immunogenicity, countering recent assertions that immune responses to this platform are inherently short-lived. Along with the phase 1 study findings [16], these results further support the capacity of mRNA vaccine design to incorporate and effectively express multiple antigens. In particular, the inclusion of the pentamer antigen underscores the ability of mRNA-based vaccines to encode the individual components of complex macromolecular structures, which are subsequently expressed, assembled, and presented in a manner that recapitulates the processes observed during natural CMV infection.

The strengths of the phase 2 Primary Trial of mRNA-1647 reported herein include its randomized, observer-blind, placebo-controlled design; the enrollment of both CMV-seronegative and CMV-seropositive participants which enabled evaluation of serostatus-specific safety and immunogenicity; and the long-term (4.5 years; through Month 54) follow-up of immunogenicity. Limitations include the lack of power to assess meaningful clinical or biologic outcomes, such as CMV seroconversion (see Table S6), with small sample sizes necessitating the reliance on descriptive analyses. Additionally, enrollment of US adults aged 18 to 40 years narrows generalizability.

## 5. Conclusions

In conclusion, this phase 2 dose-finding trial demonstrated that mRNA-1647 had an acceptable safety profile, was generally well tolerated, and induced antigen-specific antibody-mediated immune responses at all dose levels in both CMV-seronegative and CMV-seropositive adults. Long-term follow-up through 54 months provides one of the more extended durability assessments reported to date for an investigational mRNA vaccine, characterizing the trajectory of neutralizing and binding antibody responses beyond the early post-peak period and into a maintenance phase. These findings supported the implementation of the 100 μg dose level in the phase 3 pivotal efficacy trial of the investigational mRNA-1647 CMV vaccine in a global cohort of participants.

## Figures and Tables

**Table 1:** Participant baseline and demographic data ^a^.

	CMV-Seronegative					CMV-Seropositive				
		mRNA-1647					mRNA-1647			
	Placebo (*n* = 53)	50 μg (*n* = 45)	100 μg (*n* = 72)	150 μg (*n* = 45)	Overall (*n* = 215)	Placebo (*n* = 27)	50 μg (*n* = 18)	100 μg (*n* = 37)	150 μg (*n* = 18)	Overall (*n* = 100)
Sex, *n* (%)										
Male	18 (34.0)	24 (53.3)	23 (31.9)	21 (46.7)	86 (40.0)	4 (14.8)	6 (33.3)	7 (18.9)	6 (33.3)	23 (23.0)
Female	35 (66.0)	21 (46.7)	49 (68.1)	24 (53.3)	129 (60.0)	23 (85.2)	12 (66.7)	30 (81.1)	12 (66.7)	77 (77.0)
Mean age (SD), y	27.8 (6.6)	29.6 (7.1)	28.5 (6.8)	28.7 (6.1)	28.6 (6.7)	28.2 (6.5)	34.3 (4.5)	31.0 (6.6)	33.1 (6.4)	31.2 (6.5)
Race, *n* (%)										
White	45 (84.9)	39 (86.7)	63 (87.5)	37 (82.2)	184 (85.6)	24 (88.9)	15 (83.3)	28 (75.7)	14 (77.8)	81 (81.0)
Black/African American	5 (9.4)	6 (13.3)	7 (9.7)	3 (6.7)	21 (9.8)	1 (3.7)	2 (11.1)	7 (18.9)	2 (11.1)	12 (12.0)
Asian	2 (3.8)	0	0	2 (4.4)	4 (1.9)	2 (7.4)	0	0	0	2 (2.0)
American Indian or Alaska Native	0	0	0	1 (2.2)	1 (0.5)	0	0	0	0	0
Native Hawaiian or Other Pacific Islander	0	0	1 (1.4)	0	1 (0.5)	0	0	1 (2.7)	0	1 (1.0)
Multiracial	1 (1.9)	0	1 (1.4)	1 (2.2)	3 (1.4)	0	1 (5.6)	1 (2.7)	2 (11.1)	4 (4.0)
Not Reported	0	0	0	1 (2.2)	1 (0.5)	0	0	0	0	0
Ethnicity, *n* (%)										
Not Hispanic or Latino	43 (81.1)	40 (88.9)	59 (81.9)	34 (75.6)	176 (81.9)	19 (70.4)	17 (94.4)	30 (81.1)	17 (94.4)	83 (83.0)
Hispanic or Latino	10 (18.9)	5 (11.1)	12 (16.7)	11 (24.4)	38 (17.7)	8 (29.6)	1 (5.6)	7 (18.9)	1 (5.6)	17 (17.0)
Not Reported	0	0	1 (1.4)	0	1 (0.5)	0	0	0	0	0
Mean weight, kg	76.6	83.6	79.8	76.1	79.1	75.9	78.0	77.6	84.6	78.5
CMV serostatus at screening, *n* (%)										
Negative	55 (68.8)	45 (71.4)	73 (67.0)	45 (71.4)	218 (69.2)	-	-	-	-	-
Positive	25 (31.3)	18 (28.6)	36 (33.0)	18 (28.6)	97 (30.8)	-	-	-	-	-
CMV serostatus at baseline, *n* (%) ^b^										
Negative	53 (66.3)	45 (71.4)	72 (66.1)	45 (71.4)	215 (68.3)	-	-	-	-	-
Positive	27 (33.8)	18 (28.6)	37 (33.9)	18 (28.6)	100 (31.7)	-	-	-	-	-

CMV, cytomegalovirus; LLOQ, lower limit of quantification; nAb, neutralizing antibody; SD, standard deviation. ^a^ Percentages are based on the number of participants in the safety set. ^b^ Baseline CMV serology was considered positive if a participant was seronegative at screening and either the nAb titer against epithelial cell or fibroblast infection was above the LLOQ at baseline; otherwise, CMV serostatus at baseline is defined as the participant’s CMV serostatus at screening.

## Data Availability

Access to patient-level data presented in this article and supporting clinical documents with external researchers who provide methodologically sound scientific proposals will be available upon reasonable request for **products or indications that have been approved by regulators in the relevant markets and subject to review**. Such requests can be made to Moderna Inc., 325 Binney Street, Cambridge, MA, 02142 USA <<data_sharing@modernatx.com>>. A materials transfer and/or data access agreement with the sponsor will be required for accessing shared data. All other relevant data are presented in the paper. The protocol is available online at ClinicalTrials.gov (NCT04232280 and NCT04975893).

## Supplementary Materials

The following supporting information can be downloaded at: https://www.mdpi.com/article/10.3390/vaccines14050444/s1, Primary Trial Inclusion and Exclusion Criteria; Data Analysis Sets; Extension Trial Eligibility Criteria; Monitoring of CMV seroconversion; Table S1: Baseline demographics of participants by CMV serostatus (extension trial safety set); Table S2: Unsolicited treatment-emergent adverse events by CMV serostatus throughout the Primary Trial; Table S3: Summary of Unsolicited AE up to End of Primary Extension Phase by System Organ Class and Preferred Term—Primary Extension Phase Safety Set; Figure S1: Trial design; Figure S2; Solicited local adverse reactions in CMV-seronegative participants by vaccination group and grade following each dose; Figure S3: Solicited local adverse reactions in CMV-seropositive participants by vaccination group and grade following each dose; Figure S4: Solicited systemic adverse reactions in CMV-seronegative participants by vaccination group and grade following each dose; Figure S5: Solicited systemic adverse reactions in CMV-seropositive participants by vaccination group and grade following each dose; Table S4: Neutralizing and binding antibody titers after vaccination with mRNA-1647 by CMV serostatus through Month 18 (Primary Trial; per-protocol set for antibody-mediated immunogenicity); Table S5: Neutralizing and binding antibody titers after vaccination with mRNA-1647 by CMV serostatus through Month 54 (Extension Trial; per-protocol set for antibody-mediated immunogenicity); Table S6: Incidence Rate of CMV Seroconversion in CMV Seronegative Participants in Primary Extension Phase Per-Protocol Set for CMV Seroconversion.
