# Supplementary material for: Safety and Immunogenicity of the Cytomegalovirus Vaccine mRNA-1647 in Healthy Adults: Results from a Phase 2, Randomized, Controlled, Dose-Finding Trial with Long-Term Extension Follow-Up Through Month 48
Source: Vaccines (Basel). 2026 May 16;14(5):444. doi: 10.3390/vaccines14050444 (PMC13211590; doi:10.3390/vaccines14050444)
Supplement: Supplementary file 1 [file vaccines-14-00444-s001.zip › vaccines-4267722-supplementary.pdf]

**Safety and immunogenicity of the cytomegalovirus vaccine mRNA-1647 in healthy adults: results from a phase 2, randomized, controlled, dose-finding trial with long-term extension follow-up through Month 48**

## SUPPLEMENTARY MATERIALS

## METHODS

### Primary Trial Inclusion and Exclusion Criteria

Participants were eligible to participate in the trial if they met the following criteria:

1. Age and sex:
   a. Part 1: Male or female 18–40 years of age at the time of consent.
   b. Part 2: Female 18–40 years of age at the time of consent.
2. Understood and agreed to comply with trial procedures and provided written informed consent.
3. According to the assessment of the Investigator, was in good general health and capable of complying with trial procedures.
4. Had a body mass index of 18–35 kg/$m^2$.
5. Female participants of nonchildbearing potential, defined as surgically sterile (history of bilateral tubal ligation, bilateral oophorectomy, hysterectomy) or postmenopausal (amenorrhea for ≥1 year prior to screening without an alternative medical cause).
6. Female participants of childbearing age could be enrolled in the trial if the participant: 1) had a negative pregnancy test at screening and on the day of first vaccination, and 2) had practiced adequate contraception or has abstained from all activities that could result in pregnancy for ≥28 days prior to the first vaccination, and 3) had agreed to continue adequate contraception through 3 months following the last vaccination, and 4) was not currently breastfeeding.
7. Male participants agreed to practice adequate contraception from the time of the first vaccination and through 3 months after the last vaccination.

Exclusion criteria included:

1. Acute illness or febrile (temperature ≥38.0 °C/100.4 °F) on the day of the first vaccination.
2. Received any prior cytomegalovirus vaccine.
3. Positive for hepatitis B virus surface antigen, hepatitis C virus antibody, or human immunodeficiency virus (HIV) type 1 or 2 antibodies at screening.
4. Screening coagulation tests (prothrombin time or partial thromboplastin time) with a toxicity grade of ≥1.
5. Other screening laboratory results with a toxicity score grade ≥2.
6. Diagnosis or condition that, in the judgment of Investigator, is clinically unstable or may affect participant safety, assessment of safety endpoints, assessment of immune response, or adherence to trial procedures, including:
   a. Congenital or acquired immunodeficiency (including HIV infection)
   b. Diagnosed or suspected immunosuppressive condition or immune-mediated disease
   c. Chronic hepatitis
   d. Dermatologic conditions that could affect local solicited adverse reaction assessments
   e. History of anaphylaxis, urticaria, or other significant reaction requiring medical intervention after receipt of a vaccine
   f. History of bleeding disorder that is considered a contraindication to intramuscular injection or phlebotomy
   g. History of malignancy within the previous 10 years (excluding nonmelanoma skin cancer)

h. Any psychiatric or occupational condition that, in the opinion of the Investigator, might pose an additional risk due to participation in the trial or can interfere with the interpretation of trial results

7. Received or planned to receive a vaccine ≤28 days prior to the first vaccination or plans to receive a nontrial vaccine within 28 days prior to or after any trial vaccination, except for any licensed influenza vaccine, which can be administered >14 days before or after any trial vaccination. Coronavirus disease 19 (COVID-19) vaccines (regardless of manufacturer) may be administered >7 days but preferably >14 days before or after any trial vaccination, with the intention of prioritizing COVID-19 vaccination over all other considerations.
8. Received systemic immunosuppressants or immune-modifying drugs for >14 days in total within 6 months prior to the day of enrollment (for corticosteroids, ≥20 mg/day of prednisone equivalent).
9. Received intravenous immunoglobulins or plasma products within 3 months prior to the day of the first trial vaccination.
10. Part 1 participants only: Previous receipt of medications in lipid nanoparticle formulation.
11. Has donated ≥450 mL of blood products within 28 days of the Screening visit.
12. Participated in an interventional clinical trial within 28 days prior to the day of enrollment or planned to do so while enrolled in this trial.
13. Was an immediate family member or household member of trial personnel.

**Data Analysis Sets**
The following analysis sets were defined herein:

- **The randomized set**, consisting of all participants who were randomized in the trial, regardless of the participant's vaccination status in the trial; participants were included in the vaccination group to which they were randomized
- **The safety set**, consisting of all randomized participants who received any trial injection, was used for analysis of safety data except for solicited ARs; participants were included in the vaccination group corresponding to the trial injection they received
- **The solicited safety set**, consisting of all randomized participants who received any trial injection and contributed any solicited adverse reaction data, was used for the analyses of solicited adverse reactions; participants were included in the vaccination group corresponding to the trial injection they received
- **The full analysis set for antibody-mediated immunogenicity**, consisting of all randomized participants who received trial injection, had baseline (day 1) antibody-mediated immunogenicity data available for those analyses that require baseline data, had ≥1 post-injection antibody-mediated immunogenicity assessment for the analysis endpoint; participants were included in the vaccination group to which they were randomized
- **The per-protocol set for antibody-mediated immunogenicity**, defined as all of the participants of the full analysis set for antibody-mediated immunogenicity who complied with the trial injection schedule, complied with the timings of immunogenicity blood sampling to have post-injection results available for ≥1 assay component corresponding to the immunogenicity analysis objective, and had no major protocol deviations that impacted immune response during the period corresponding to the immunogenicity analysis objective (participants who were CMV-seronegative at screening and became CMV-seropositive before or on the day of dose 1 were excluded from this set), served as the primary population for the analysis of antibody-mediated immunogenicity data; participants were included in the vaccination group to which they were randomized

**Extension Trial Eligibility Criteria**

*Eligibility Criteria*

Male and female participants were eligible for inclusion if the following criteria applied:

1. CMV-seronegative at the primary phase 2 trial screening visit, completed the final study P202 visit, and remained CMV-seronegative at the phase 2 extension trial screening visit
2. CMV-seropositive at the primary phase 2 trial screening visit, were randomly assigned to receive mRNA-1647 (not placebo), and completed the final visit in the primary phase 2 trial
3. Understood and agreed to comply with the trial procedures and provided written informed consent
4. Was in good general health according to the assessment of the investigator and was able to comply with trial procedures

Male and female participants were not eligible for inclusion if any of the following criteria applied:

1. Received any CMV vaccine other than mRNA-1647
2. Had a diagnosis or condition that, in the judgment of the investigator, may affect participant safety, assessment of safety endpoints, assessment of immune response, or adherence to trial procedures, including any medical, psychiatric, or occupational condition that in the opinion of the investigator might pose additional risk due to participation in the trial or could interfere with the interpretation of any trial results

Participation in interventional or non-interventional trials may be permitted during the extension trial.

**Monitoring of CMV seroconversion**

The presence of primary CMV infection was monitored throughout the extension trial as an indicator of seroconversion among CMV-seronegative participants, with serum samples collected every 6 months for this purpose. Seroconversion due to primary CMV infection is defined if a CMV-seronegative participant obtains a positive result for the detection of serum IgG against ≥1 or 4 recombinant CMV antigens that are not encoded by mRNA-1647 (pp150, pp28 pp52, pp38), as measured by a platform-based automated immunoassay.

**Table S1. Baseline demographics of participants by CMV serostatus (extension trial safety set)**

| | CMV-seronegative | | | | | CMV-seropositive | | | |
|---|---|---|---|---|---|---|---|---|---|
| | | mRNA-1647 | | | | mRNA-1647 | | | |
| | Placebo (*n*=26) | 50 µg (*n*=18) | 100 µg (*n*=31) | 150 µg (*n*=24) | Total (*n*=73) | 50 µg (*n*=11) | 100 µg (*n*=16) | 150 µg (*n*=9) | Total (*n*=36) |
| **Age, mean (SD), years** | 31.6 (7.0) | 32.9 (6.3) | 29.2 (6.8) | 30.8 (6.4) | 30.6 (6.6) | 36.4 (5.5) | 32.3 (6.4) | 37.0 (5.4) | 34.7 (6.1) |
| **Sex, *n* (%)** | | | | | | | | | |
| Male | 8 (30.8) | 7 (38.9) | 8 (25.8) | 12 (50.0) | 27 (37.0) | 5 (45.5) | 2 (12.5) | 4 (44.4) | 11 (30.6) |
| Female | 18 (69.2) | 11 (61.1) | 23 (74.2) | 12 (50.0) | 46 (63.0) | 6 (54.5) | 14 (87.5) | 5 (55.6) | 25 (69.4) |
| **Race, *n* (%)** | | | | | | | | | |
| White | 24 (92.3) | 14 (77.8) | 29 (93.5) | 21 (87.5) | 64 (87.7) | 10 (90.9) | 12 (75.0) | 7 (77.8) | 29 (80.6) |
| Black | 1 (3.8) | 4 (22.2) | 2 (6.5) | 0 | 6 (8.2) | 0 | 3 (18.8) | 0 | 3 (8.3) |
| Asian | 1 (3.8) | 0 | 0 | 1 (4.2) | 1 (1.4) | 0 | 0 | 0 | 0 |
| Native Hawaiian or Pacific Islander | 0 | 0 | 0 | 0 | 0 | 0 | 1 (6.3) | 0 | 1 (2.8) |
| Multiracial | 0 | 0 | 0 | 1 (4.2) | 1 (1.4) | 1 (9.1) | 0 | 2 (22.2) | 3 (8.3) |
| NR | 0 | 0 | 0 | 1 (4.2) | 1 (1.4) | 0 | 0 | 0 | 0 |
| **Ethnicity, *n* (%)** | | | | | | | | | |
| Not Hispanic or Latino | 23 (88.5) | 17 (94.4) | 27 (87.1) | 19 (79.2) | 63 (86.3) | 11 (100.0) | 12 (75.0) | 8 (88.9) | 31 (86.1) |
| Hispanic or Latino | 3 (11.5) | 1 (5.6) | 4 (12.9) | 5 (20.8) | 10 (13.7) | 0 | 4 (25.0) | 1 (11.1) | 5 (13.9) |
| **Primary Trial injections received[a]** | | | | | | | | | |
| Injections 1, 2, and 3 | 25 (96.2) | 17 (94.4) | 29 (93.5) | 19 (79.2) | 65 (89.0) | 11 (100.0) | 14 (87.5) | 9 (100.0) | 34 (94.4) |
| Injections 1 and 2 only | 1 (3.8) | 1 (5.6) | 2 (6.5) | 4 (16.7) | 7 (9.6) | 0 | 2 (12.5) | 0 | 2 (5.6) |

| | CMV-seronegative | | | | | CMV-seropositive | | | |
|---|---|---|---|---|---|---|---|---|---|
| | | mRNA-1647 | | | | mRNA-1647 | | | |
| | Placebo (*n*=26) | 50 µg (*n*=18) | 100 µg (*n*=31) | 150 µg (*n*=24) | Total (*n*=73) | 50 µg (*n*=11) | 100 µg (*n*=16) | 150 µg (*n*=9) | Total (*n*=36) |
| Injection 1 only | 0 | 0 | 0 | 1 (4.2) | 1 (1.4) | 0 | 0 | 0 | 0 |

Abbreviations: CMV, cytomegalovirus; SD, standard deviation; NR, not reported.

[a] Data are representative of the extension phase safety set consisting of participants enrolled in the extension trial who were included in the primary phase 2 trial safety set (all randomized participants who received any injection in the primary phase 2 trial).

**Table S2. Unsolicited treatment-emergent adverse events by CMV serostatus throughout the Primary Trial**

| | CMV-seronegative, *n* (%) | | | | | CMV-seropositive, *n* (%) | | | | |
|---|---|---|---|---|---|---|---|---|---|---|
| | | mRNA-1647 | | | | | mRNA-1647 | | | |
| | Placebo (*n* = 53) | 50 µg (*n* = 45) | 100 µg (*n* = 72) | 150 µg (*n* = 45) | Total (*n* = 162) | Placebo (*n* = 27) | 50 µg (*n* = 18) | 100 µg (*n* = 37) | 150 µg (*n* = 18) | Total (*n* = 73) |
| Any unsolicited TEAEs | | | | | | | | | | |
| All | 25 (47.2) | 19 (42.2) | 35 (48.6) | 20 (44.4) | 74 (45.7) | 11 (40.7) | 10 (55.6) | 18 (48.6) | 6 (33.3) | 34 (46.6) |
| Serious | 0 | 0 | 1 (1.4) | 0 | 1 (0.6) | 0 | 0 | 1 (2.7) | 0 | 1 (1.4) |
| Fatal | 0 | 0 | 0 | 0 | 0 | 0 | 0 | 0 | 0 | 0 |
| MAAE | 19 (35.8) | 13 (28.9) | 24 (33.3) | 14 (31.1) | 51 (31.5) | 9 (33.3) | 5 (27.8) | 11 (29.7) | 1 (5.6) | 17 (23.3) |
| Leading to discontinuation of trial dose | 0 | 1 (2.2) | 2 (2.8) | 0 | 3 (1.9) | 1 (3.7) | 0 | 2 (5.4) | 0 | 2 (2.7) |
| Severe (grade 3 or higher) | 7 (13.2) | 9 (20.0) | 18 (25.0) | 3 (6.7) | 30 (18.5) | 3 (11.1) | 4 (22.2) | 5 (13.5) | 0 | 9 (12.3) |
| TEAEs related to vaccination | | | | | | | | | | |
| All | 3 (5.7) | 1 (2.2) | 12 (16.7) | 2 (4.4) | 15 (9.3) | 0 | 2 (11.1) | 2 (5.4) | 4 (22.2) | 8 (11.0) |
| Serious | 0 | 0 | 0 | 0 | 0 | 0 | 0 | 0 | 0 | 0 |
| Fatal | 0 | 0 | 0 | 0 | 0 | 0 | 0 | 0 | 0 | 0 |
| MAAE | 2 (3.8) | 1 (2.2) | 5 (6.9) | 2 (4.4) | 8 (4.9) | 0 | 1 (5.6) | 1 (2.7) | 0 | 2 (2.7) |
| Leading to discontinuation of trial dose | 0 | 0 | 1 (1.4) | 0 | 1 (0.6) | 0 | 0 | 1 (2.7) | 0 | 1 (1.4) |
| Severe (grade 3 or higher) | 0 | 0 | 2 (2.8) | 0 | 2 (1.2) | 0 | 0 | 1 (2.7) | 0 | 1 (1.4) |

CMV, cytomegalovirus; MAAE, medically attended adverse event; TEAE, treatment-emergent adverse event.
Data are representative of the safety set.

## Table S3. Summary of Unsolicited AE up to End of Primary Extension Phase by System Organ Class and Preferred Term – Primary Extension Phase Safety Set

| | CMV-Seronegative | | | | | | CMV-Seropositive | | | | Overall | | | | | |
|---|---|---|---|---|---|---|---|---|---|---|---|---|---|---|---|---|
| | | mRNA-1647 | | | | | mRNA-1647 | | | | | mRNA-1647 | | | | |
| System Organ Class<br>Preferred Term | Placebo<br>(N=26)<br>n (%) | 50 µg<br>(N=18)<br>n (%) | 100 µg<br>(N=31)<br>n (%) | 150 µg<br>(N=24)<br>n (%) | Total<br>(N=73)<br>n (%) | Overall<br>(N=99)<br>n (%) | 50 µg<br>(N=11)<br>n (%) | 100 µg<br>(N=16)<br>n (%) | 150 µg<br>(N=9)<br>n (%) | Total<br>(N=36)<br>n (%) | Placebo<br>(N=26)<br>n (%) | 50 µg<br>(N=29)<br>n (%) | 100 µg<br>(N=47)<br>n (%) | 150 µg<br>(N=33)<br>n (%) | Total<br>(N=109)<br>n (%) | Overall<br>(N=135)<br>n (%) |
| Number of Participants Reporting Unsolicited Adverse Events | 3 (11.5) | 0 | 5 (16.1) | 2 (8.3) | 7 (9.6) | 10 (10.1) | 0 | 1 (6.3) | 0 | 1 (2.8) | 3 (11.5) | 0 | 6 (12.8) | 2 (6.1) | 8 (7.3) | 11 (8.1) |
| Number of Unsolicited Adverse Events | 3 | 0 | 11 | 3 | 14 | 17 | 0 | 1 | 0 | 1 | 3 | 0 | 12 | 3 | 15 | 18 |
| Infections and infestations | 0 | 0 | 2 (6.5) | 0 | 2 (2.7) | 2 (2.0) | 0 | 1 (6.3) | 0 | 1 (2.8) | 0 | 0 | 3 (6.4) | 0 | 3 (2.8) | 3 (2.2) |
| Bacteraemia | 0 | 0 | 1 (3.2) | 0 | 1 (1.4) | 1 (1.0) | 0 | 0 | 0 | 0 | 0 | 0 | 1 (2.1) | 0 | 1 (0.9) | 1 (0.7) |
| Escherichia bacteraemia | 0 | 0 | 1 (3.2) | 0 | 1 (1.4) | 1 (1.0) | 0 | 0 | 0 | 0 | 0 | 0 | 1 (2.1) | 0 | 1 (0.9) | 1 (0.7) |
| Escherichia pyelonephritis | 0 | 0 | 1 (3.2) | 0 | 1 (1.4) | 1 (1.0) | 0 | 0 | 0 | 0 | 0 | 0 | 1 (2.1) | 0 | 1 (0.9) | 1 (0.7) |
| Sepsis | 0 | 0 | 1 (3.2) | 0 | 1 (1.4) | 1 (1.0) | 0 | 0 | 0 | 0 | 0 | 0 | 1 (2.1) | 0 | 1 (0.9) | 1 (0.7) |
| Urinary tract infection | 0 | 0 | 0 | 0 | 0 | 0 | 0 | 1 (6.3) | 0 | 1 (2.8) | 0 | 0 | 1 (2.1) | 0 | 1 (0.9) | 1 (0.7) |
| Psychiatric disorders | 0 | 0 | 1 (3.2) | 0 | 1 (1.4) | 1 (1.0) | 0 | 0 | 0 | 0 | 0 | 0 | 1 (2.1) | 0 | 1 (0.9) | 1 (0.7) |
| Suicidal ideation | 0 | 0 | 1 (3.2) | 0 | 1 (1.4) | 1 (1.0) | 0 | 0 | 0 | 0 | 0 | 0 | 1 (2.1) | 0 | 1 (0.9) | 1 (0.7) |
| Nervous system disorders | 0 | 0 | 1 (3.2) | 1 (4.2) | 2 (2.7) | 2 (2.0) | 0 | 0 | 0 | 0 | 0 | 0 | 1 (2.1) | 1 (3.0) | 2 (1.8) | 2 (1.5) |
| Nerve compression | 0 | 0 | 1 (3.2) | 0 | 1 (1.4) | 1 (1.0) | 0 | 0 | 0 | 0 | 0 | 0 | 1 (2.1) | 0 | 1 (0.9) | 1 (0.7) |
| Seizure | 0 | 0 | 0 | 1 (4.2) | 1 (1.4) | 1 (1.0) | 0 | 0 | 0 | 0 | 0 | 0 | 0 | 1 (3.0) | 1 (0.9) | 1 (0.7) |
| Trigeminal neuralgia | 0 | 0 | 1 (3.2) | 0 | 1 (1.4) | 1 (1.0) | 0 | 0 | 0 | 0 | 0 | 0 | 1 (2.1) | 0 | 1 (0.9) | 1 (0.7) |
| Gastrointestinal disorders | 1 (3.8) | 0 | 0 | 0 | 0 | 1 (1.0) | 0 | 0 | 0 | 0 | 1 (3.8) | 0 | 0 | 0 | 0 | 1 (0.7) |
| Upper gastrointestinal haemorrhage | 1 (3.8) | 0 | 0 | 0 | 0 | 1 (1.0) | 0 | 0 | 0 | 0 | 1 (3.8) | 0 | 0 | 0 | 0 | 1 (0.7) |
| Hepatobiliary disorders | 0 | 0 | 1 (3.2) | 0 | 1 (1.4) | 1 (1.0) | 0 | 0 | 0 | 0 | 0 | 0 | 1 (2.1) | 0 | 1 (0.9) | 1 (0.7) |
| Hepatitis alcoholic | 0 | 0 | 1 (3.2) | 0 | 1 (1.4) | 1 (1.0) | 0 | 0 | 0 | 0 | 0 | 0 | 1 (2.1) | 0 | 1 (0.9) | 1 (0.7) |
| Musculoskeletal and connective tissue disorders | 0 | 0 | 0 | 1 (4.2) | 1 (1.4) | 1 (1.0) | 0 | 0 | 0 | 0 | 0 | 0 | 0 | 1 (3.0) | 1 (0.9) | 1 (0.7) |
| Osteochondral defects | 0 | 0 | 0 | 1 (4.2) | 1 (1.4) | 1 (1.0) | 0 | 0 | 0 | 0 | 0 | 0 | 0 | 1 (3.0) | 1 (0.9) | 1 (0.7) |
| Pregnancy, puerperium and perinatal conditions | 2 (7.7) | 0 | 1 (3.2) | 0 | 1 (1.4) | 3 (3.0) | 0 | 0 | 0 | 0 | 2 (7.7) | 0 | 1 (2.1) | 0 | 1 (0.9) | 3 (2.2) |
| Abortion spontaneous | 2 (7.7) | 0 | 1 (3.2) | 0 | 1 (1.4) | 3 (3.0) | 0 | 0 | 0 | 0 | 2 (7.7) | 0 | 1 (2.1) | 0 | 1 (0.9) | 3 (2.2) |
| General disorders and administration site conditions | 0 | 0 | 1 (3.2) | 0 | 1 (1.4) | 1 (1.0) | 0 | 0 | 0 | 0 | 0 | 0 | 1 (2.1) | 0 | 1 (0.9) | 1 (0.7) |
| Chest discomfort | 0 | 0 | 1 (3.2) | 0 | 1 (1.4) | 1 (1.0) | 0 | 0 | 0 | 0 | 0 | 0 | 1 (2.1) | 0 | 1 (0.9) | 1 (0.7) |
| Injury, poisoning and procedural complications | 0 | 0 | 1 (3.2) | 0 | 1 (1.4) | 1 (1.0) | 0 | 0 | 0 | 0 | 0 | 0 | 1 (2.1) | 0 | 1 (0.9) | 1 (0.7) |
| Upper limb fracture | 0 | 0 | 1 (3.2) | 0 | 1 (1.4) | 1 (1.0) | 0 | 0 | 0 | 0 | 0 | 0 | 1 (2.1) | 0 | 1 (0.9) | 1 (0.7) |

AE = Adverse Event.

The summarized adverse event is for any event not present before the date of informed consent or any event already present that worsens in intensity or frequency on or after the date of informed consent.
For analysis with a data cutoff, this table includes adverse events up to end of primary extension phase or data cutoff date, whichever occurred earlier.
Percentages are based on the number of participants in the Safety Set. MedDRA version 28.0.

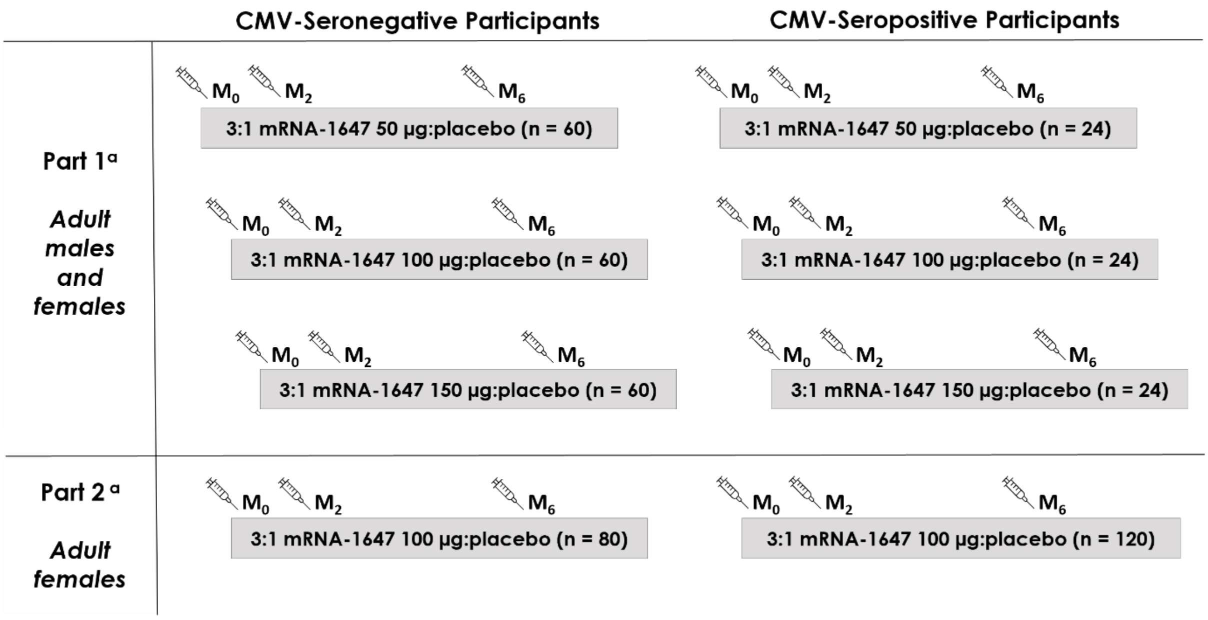


**Figure S1. Trial design. CMV, cytomegalovirus; M, month.**

[a] Data from Parts 1 and 2 were combined.

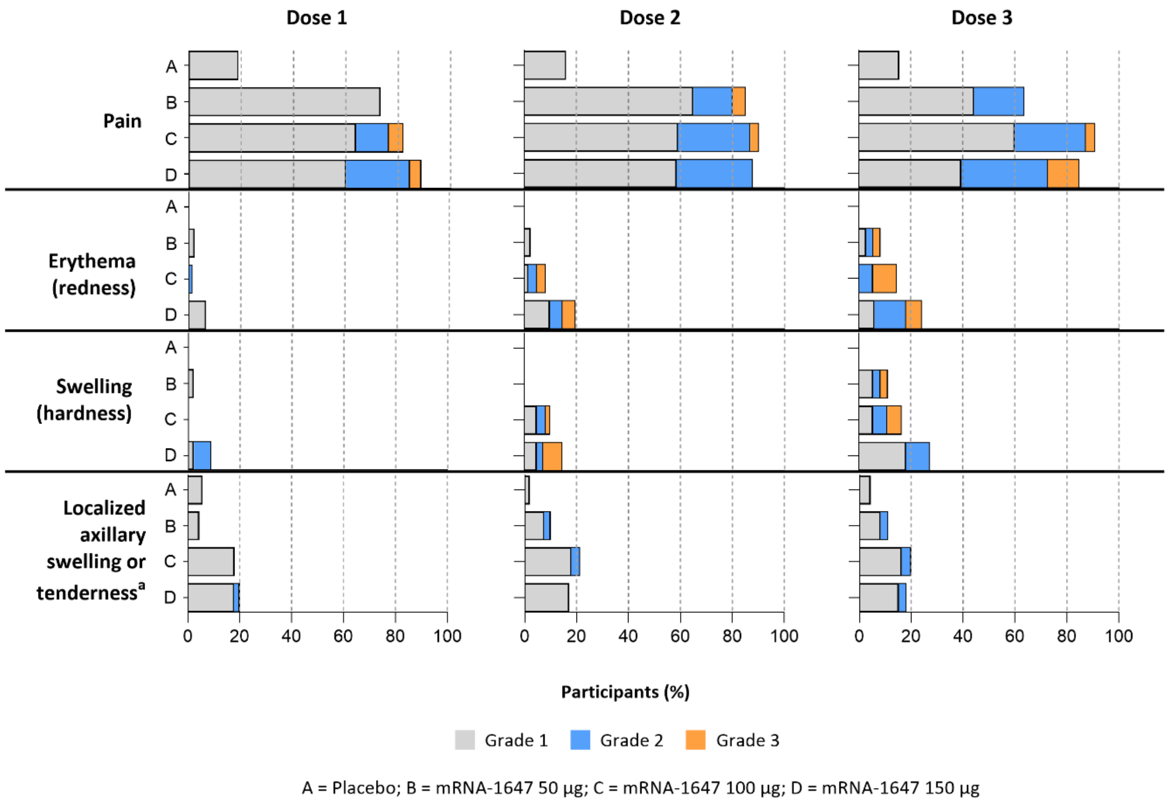


**Figure S2. Solicited local adverse reactions in CMV-seronegative participants by vaccination group and grade following each dose. Data are from doses 1 (*n* = 215), 2 (*n* = 192), and 3 (*n* = 169) solicited safety sets. As depicted in the key, values for grades 1 (grey bars), 2 (blue bars), and 3 (orange bars) are shown for placebo (A rows), mRNA-1647 50 µg (B rows), mRNA-1647 100 µg (C rows), and mRNA-1647 150 µg (D rows). CMV, cytomegalovirus.**

[a] Ipsilateral to vaccination site.

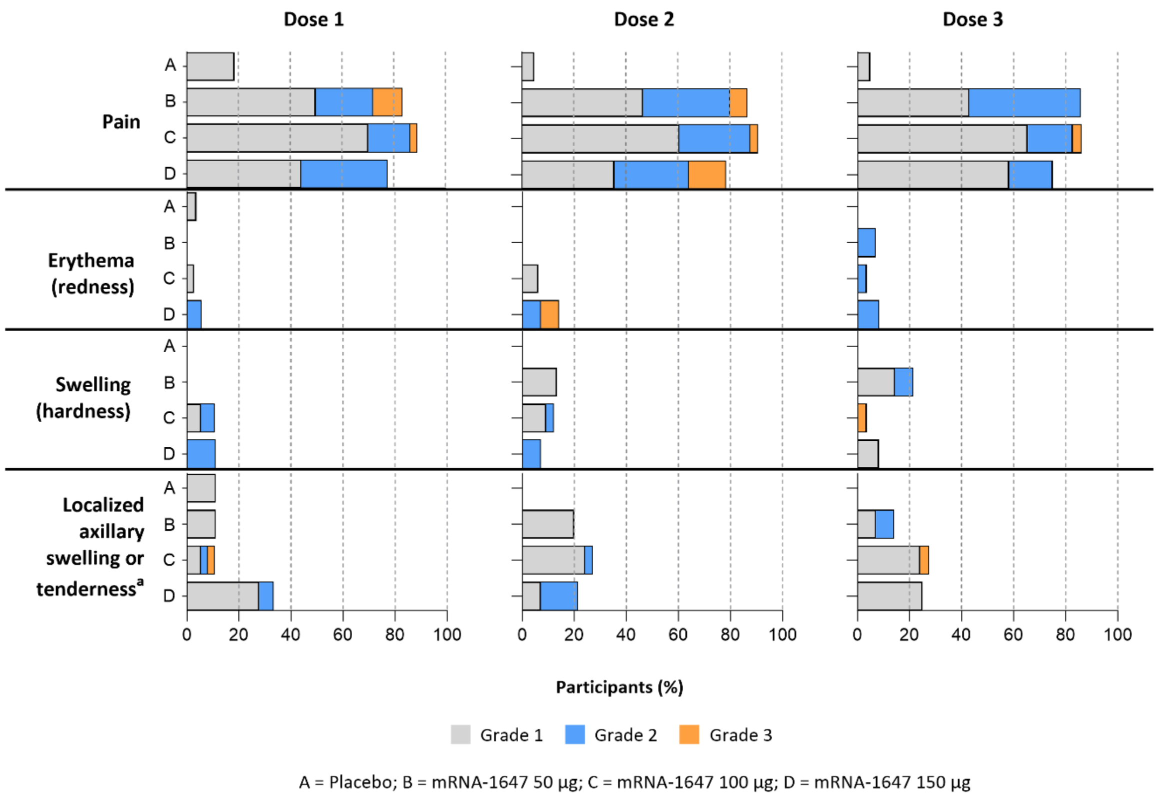


**Figure S3. Solicited local adverse reactions in CMV-seropositive participants by vaccination group and grade following each dose. Data are from doses 1 (*n* = 100), 2 (*n* = 83), and 3 (*n* = 76) solicited safety sets. As depicted in the key, values for grades 1 (grey bars), 2 (blue bars), and 3 (orange bars) are shown for placebo (A rows), mRNA-1647 50 µg (B rows), mRNA-1647 100 µg (C rows), and mRNA-1647 150 µg (D rows). CMV, cytomegalovirus.**

[a] Ipsilateral to vaccination site.

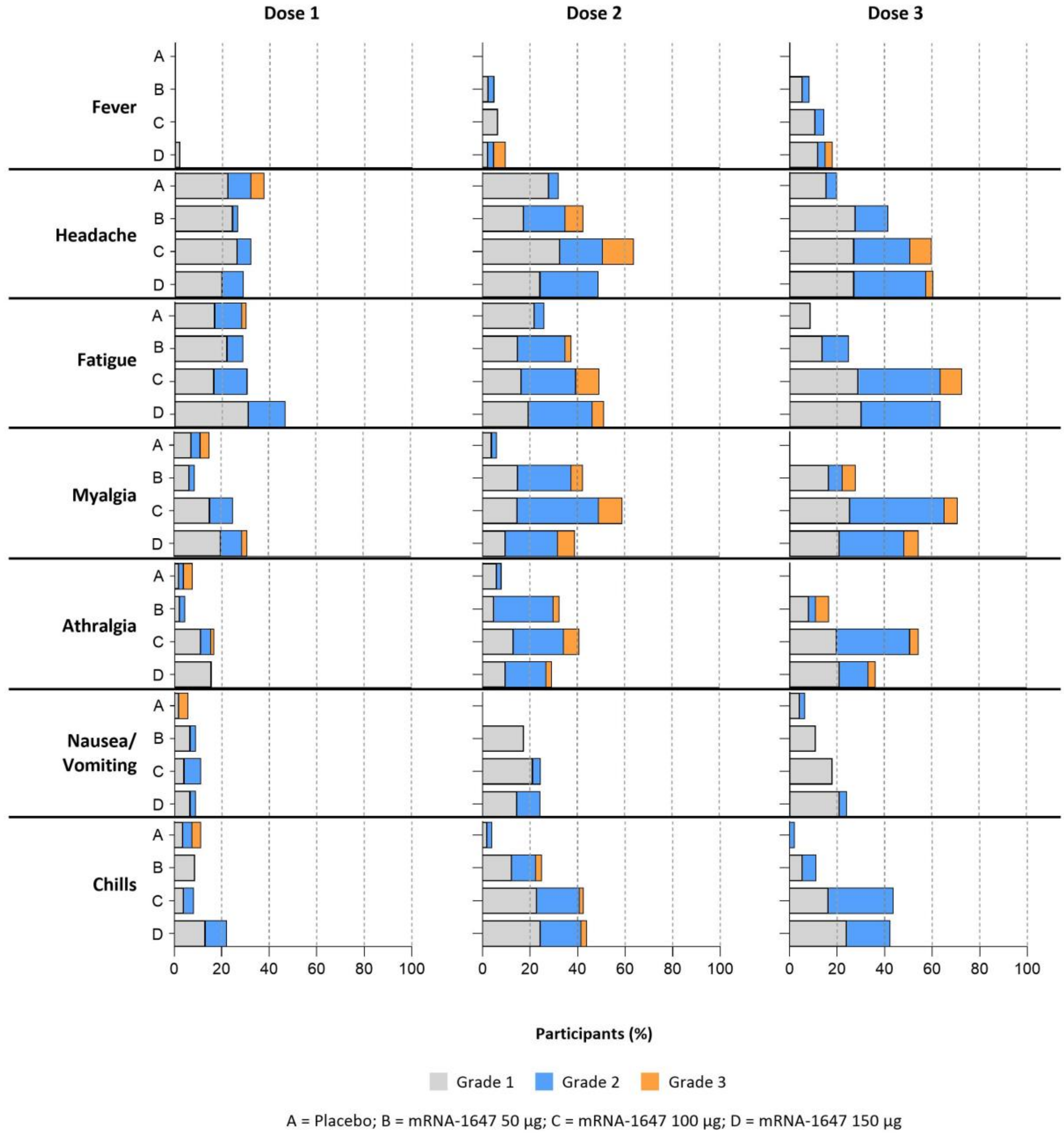


**Figure S4. Solicited systemic adverse reactions in CMV-seronegative participants by vaccination group and grade following each dose.** [a] **Data are from doses 1 (*n* = 215), 2 (*n* = 192), and 3 (*n* = 169) solicited safety sets. As depicted in the key, values for grades 1 (grey bars), 2 (blue bars), and 3 (orange bars), are shown for placebo (A rows), mRNA-1647 50 µg (B rows), mRNA-1647 100 µg (C rows), and mRNA-1647 150 µg (D rows). CMV, cytomegalovirus.**

[a] Rash was also solicited as a yes/no value: dose 1, yes (7.5%, 4.4%, 6.9%, and 6.7% for placebo, mRNA-1647 50 µg, mRNA-1647 100 µg, and mRNA-1647 150 µg, respectively); dose 1, no (92.5%, 95.6%, 93.1%, and 93.3% for placebo, mRNA-1647 50 µg, mRNA-1647 100 µg, and mRNA-1647 150 µg, respectively); dose 2, yes (0%, 0%, 8.2%, and 14.6% for placebo, mRNA-1647 50 µg, mRNA-1647 100 µg, and mRNA-1647 150 µg, respectively); dose 2, no (100%, 100%, 91.8%, and 85.4% for placebo, mRNA-1647 50 µg, mRNA-1647 100 µg, and mRNA-1647 150 µg, respectively); dose 3, yes (2.2%, 0%, 12.7%, and 6.1% for placebo, mRNA-1647 50 µg, mRNA-1647 100 µg, and mRNA-1647 150 µg, respectively); and dose 3, no (97.8%, 100.0%, 87.3%, and 93.9% for placebo, mRNA-1647 50 µg, mRNA-1647 100 µg, and mRNA-1647 150 µg, respectively).

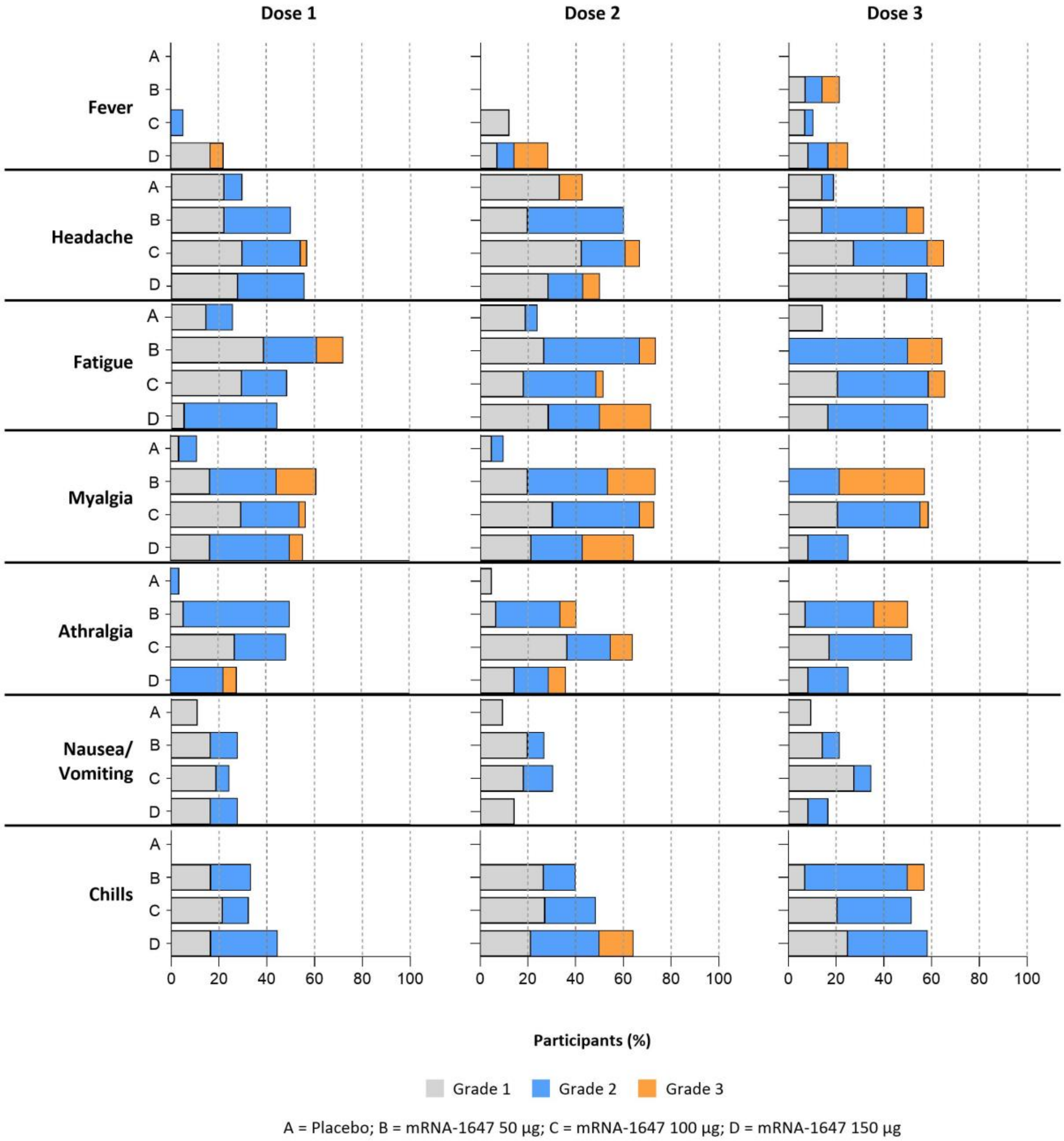


**Figure S5. Solicited systemic adverse reactions in CMV-seropositive participants by vaccination group and grade following each dose.[a] Data are from doses 1 (*n* = 100), 2 (*n* = 83), and 3 (*n* = 76) solicited safety sets. As depicted in the key, values for grades 1 (grey bars), 2 (blue bars), and 3 (orange bars) are shown for placebo (A rows), mRNA-1647 50 µg (B rows), mRNA-1647 100 µg (C rows), and mRNA-1647 150 µg (D rows). CMV, cytomegalovirus.**

[a] Rash was also solicited as a yes/no value: dose 1, yes (14.8%, 16.7%, 5.4%, and 5.6% for placebo, mRNA-1647 50 µg, mRNA-1647 100 µg, and mRNA-1647 150 µg, respectively); dose 1, no (85.2%, 83.3%, 94.6%, and 94.4% for placebo, mRNA-1647 50 µg, mRNA-1647 100 µg, and mRNA-1647 150 µg, respectively); dose 2, yes (9.5%, 6.7%, 6.1%, and 7.1% for placebo, mRNA-1647 50 µg, mRNA-1647 100 µg, and mRNA-1647 150 µg, respectively); dose 2, no (90.5%, 93.3%, 93.9%, and 92.9% for placebo, mRNA-1647 50 µg, mRNA-1647 100 µg, and mRNA-1647 150 µg, respectively); dose 3, yes (4.8%, 0%, 3.4%, and 8.3% for placebo, mRNA-1647 50 µg, mRNA-1647 100 µg, and mRNA-1647 150 µg, respectively); and dose 3, no (95.2%, 100.0%, 96.6%, and 91.7% for placebo, mRNA-1647 50 µg, mRNA-1647 100 µg, and mRNA-1647 150 µg, respectively).

**Table S4. Neutralizing and binding antibody titers after vaccination with mRNA-1647 by CMV serostatus through Month 18 (Primary Trial; per-protocol set for antibody-mediated immunogenicity)**

| | CMV-Seronegative | | | | | CMV-Seropositive | | | |
|---|---|---|---|---|---|---|---|---|---|
| | | mRNA-1647 | | | | mRNA-1647 | | | |
| Timepoint | Placebo (N=53) | 50 µg (N=44) | 100 µg (N=64) | 150 µg (N=44) | Total (N=152) | 50 µg (N=15) | 100 µg (N=34) | 150 µg (N=15) | Total (N=64) |
| **Neutralizing Antibodies Against Epithelial Cell Infection [a]** | | | | | | | | | |
| **Baseline** | | | | | | | | | |
| *n* | 53 | 44 | 64 | 44 | 152 | 15 | 34 | 15 | 64 |
| GMT (95% CI) | 8.0 NE, NE | 8.0 NE, NE | 8.0 NE, NE | 8.0 NE, NE | 8.0 NE, NE | 2250.0 (589.3, 8591.3) | 4571.9 (2708.6, 7717.2) | 7295.7 (4109.4, 12952.5) | 4320.2 (2826.0, 6604.4) |
| **Month 1** | | | | | | | | | |
| *n* | 51 | 44 | 64 | 38 | 146 | 15 | 33 | 13 | 61 |
| GMT (95% CI) | 8.9 (7.2, 11.0) | 1120.5 (684.0, 1835.6) | 1664.0 (1032.9, 2680.5) | 3802.0 (2712.8, 5328.3) | 1831.4 (1389.5, 2414.0) | 27061.6 (7391.8, 99073.1) | 77414.8 (47189.5, 126999.8) | 102921.4 (55936.7, 189371.3) | 63523.6 (41451.0, 97350.0) |
| GMFR (95% CI) | 1.11 (0.90, 1.38) | 140.06 (85.50, 229.45) | 207.99 (129.12, 335.06) | 475.24 (339.10, 666.04) | 228.93 (173.68, 301.75) | 12.03 (7.30, 19.83) | 17.13 (10.09, 29.10) | 12.82 (5.24, 31.37) | 14.76 (10.42, 20.91) |
| **Month 2** | | | | | | | | | |
| *n* | 41 | 38 | 48 | 35 | 121 | 15 | 25 | 11 | 51 |
| GMT 95% CI | 8.3 (7.7, 9.0) | 875.8 (546.9, 1402.5) | 742.0 (447.3, 1230.8) | 1814.9 (1264.2, 2605.3) | 1012.5 (771.5, 1328.7) | 30228.3 (7729.8, 118210.8) | 50596.3 (27015.5, 94759.9) | 88938.9 (50342.7, 157125.5) | 49108.9 (29931.2, 80574.4) |
| GMFR | 1.04 | 109.47 | 92.75 | 226.86 | 126.56 | 13.43 | 14.61 | 11.89 | 13.63 |

| | CMV-Seronegative | | | | | CMV-Seropositive | | | |
|---|---|---|---|---|---|---|---|---|---|
| | | mRNA-1647 | | | | mRNA-1647 | | | |
| Timepoint | Placebo (N=53) | 50 µg (N=44) | 100 µg (N=64) | 150 µg (N=44) | Total (N=152) | 50 µg (N=15) | 100 µg (N=34) | 150 µg (N=15) | Total (N=64) |
| (95% CI) | (0.96, 1.12) | (68.36, 175.31) | (55.92, 153.85) | (158.03, 325.67) | (96.43, 166.09) | (7.55, 23.90) | (8.03, 26.59) | (6.07, 23.29) | (9.64, 19.29) |
| **Month 3** | | | | | | | | | |
| *n* | 46 | 27 | 56 | 36 | 119 | 15 | 29 | 13 | 57 |
| GMT (95% CI) | 8.0 NE, NE | 61759.1 (39400.1, 96806.5) | 44745.1 (32240.9, 62098.7) | 49581.0 (36690.2, 67000.7) | 49657.3 (40590.4, 60749.5) | 104605.7 (67545.8, 161998.9) | 85821.2 (53651.2, 137280.7) | 115517.2 (72279.5, 184619.6) | 96749.5 (73584.9, 127206.3) |
| GMFR (95% CI) | 1.00 NE, NE | 7719.89 (4925.02, 12100.81) | 5593.13 (4030.12, 7762.34) | 6197.62 (4586.28, 8375.09) | 6207.16 (5073.80, 7593.69) | 46.49 (13.06, 165.46) | 22.34 (13.11, 38.05) | 17.06 (9.58, 30.37) | 25.47 (16.56, 39.18) |
| **Month 6** | | | | | | | | | |
| *n* | 32 | 32 | 36 | 28 | 96 | 14 | 19 | 10 | 43 |
| GMT (95% CI) | 9.8 (6.5, 14.8) | 8152.7 (4672.1, 14226.2) | 10780.1 (7194.4, 16152.9) | 9587.3 (7143.1, 12867.7) | 9491.4 (7416.5, 12146.6) | 41511.4 (24866.9, 69296.9) | 48035.5 (26414.1, 87355.1) | 81979.3 (50734.4, 132466.3) | 51869.1 (37732.0, 71302.9) |
| GMFR (95% CI) | 1.22 (0.81, 1.85) | 1019.09 (584.01, 1778.27) | 1347.51 (899.30, 2019.11) | 1198.41 (892.89, 1608.47) | 1186.42 (927.07, 1518.32) | 18.99 (5.32, 67.77) | 10.10 (5.21, 19.61) | 10.82 (5.68, 20.62) | 12.61 (7.70, 20.65) |
| **Month 7** | | | | | | | | | |
| *n* | 37 | 27 | 40 | 27 | 94 | 13 | 24 | 11 | 48 |
| GMT (95% CI) | 8.0 NE, NE | 117022.3 | 136935.3 | 101113.5 | 119972.6 | 87440.9 | 86359.0 | 105862.1 | 90790.0 |

| Timepoint | CMV-Seronegative | | | | | CMV-Seropositive | | | |
|---|---|---|---|---|---|---|---|---|---|
| | | mRNA-1647 | | | | mRNA-1647 | | | |
| | Placebo (N=53) | 50 µg (N=44) | 100 µg (N=64) | 150 µg (N=44) | Total (N=152) | 50 µg (N=15) | 100 µg (N=34) | 150 µg (N=15) | Total (N=64) |
| | | (71908.7, 190439.0) | (97637.3, 192050.2) | (75956.7, 134602.1) | (97209.9, 148065.5) | (49755.7, 153669.1) | (52162.3, 142974.4) | (57020.2, 196540.7) | (66884.6, 123239.5) |
| GMFR (95% CI) | 1.00 NE, NE | 14627.79 (8988.59, 23804.88) | 17116.91 (12204.67, 24006.27) | 12639.18 (9494.59, 16825.27) | 14996.58 (12151.23, 18508.18) | 40.35 (7.45, 218.58) | 17.97 (10.61, 30.44) | 12.88 (7.37, 22.49) | 20.73 (12.44, 34.53) |
| **Month 12** | | | | | | | | | |
| *n* | 34 | 27 | 39 | 27 | 93 | 14 | 18 | 12 | 44 |
| GMT (95% CI) | 9.7 (6.5, 14.4) | 20622.0 (13451.7, 31614.3) | 18790.9 (13864.4, 25467.9) | 28526.8 (18535.4, 43904.1) | 21792.6 (17628.4, 26940.5) | 38787.8 (23499.4, 64022.7) | 27083.3 (17644.7, 41570.9) | 65788.7 (37545.8, 115276.4) | 38677.5 (29211.0, 51211.9) |
| GMFR (95% CI) | 1.21 (0.82, 1.80) | 2577.75 (1681.46, 3951.78) | 2348.86 (1733.06, 3183.48) | 3565.85 (2316.93, 5488.01) | 2724.07 (2203.55, 3367.56) | 17.75 (4.83, 65.24) | 5.27 (3.02, 9.20) | 8.58 (3.99, 18.45) | 8.86 (5.39, 14.56) |
| **Month 18** | | | | | | | | | |
| *n* | 36 | 22 | 36 | 23 | 81 | 14 | 17 | 11 | 42 |
| GMT (95% CI) | 12.4 (7.0, 22.2) | 10145.3 (6364.0, 16173.2) | 11658.5 (8180.6, 16615.1) | 15762.9 (9572.3, 25957.3) | 12230.4 (9633.5, 15527.2) | 23536.1 (14454.3, 38324.3) | 18460.9 (11694.5, 29142.5) | 51490.6 (23328.5, 113649.7) | 26187.0 (19029.4, 36036.8) |
| GMFR (95% CI) | 1.55 (0.87, 2.77) | 1268.16 (795.51, 2021.65) | 1457.31 (1022.57, 2076.89) | 1970.37 (1196.53, 3244.66) | 1528.80 (1204.19, 1940.90) | 10.77 (3.16, 36.74) | 3.97 (2.29, 6.90) | 6.88 (2.90, 16.31) | 6.40 (3.92, 10.44) |

| | CMV-Seronegative | | | | | CMV-Seropositive | | | |
|---|---|---|---|---|---|---|---|---|---|
| | | mRNA-1647 | | | | mRNA-1647 | | | |
| Timepoint | Placebo (N=53) | 50 µg (N=44) | 100 µg (N=64) | 150 µg (N=44) | Total (N=152) | 50 µg (N=15) | 100 µg (N=34) | 150 µg (N=15) | Total (N=64) |
| **Neutralizing Antibodies Against Fibroblast Infection[b]** | | | | | | | | | |
| **Baseline** | | | | | | | | | |
| *n* | 53 | 44 | 64 | 44 | 152 | 15 | 34 | 15 | 64 |
| GMT (95% CI) | 8.0 NE, NE | 8.0 NE, NE | 8.0 NE, NE | 8.0 NE, NE | 8.0 NE, NE | 2507.4 (651.1, 9655.4) | 4837.6 (2865.2, 8167.7) | 6358.9 (3624.1, 11157.7) | 4421.5 (2902.5, 6735.4) |
| **Month 1** | | | | | | | | | |
| *n* | 51 | 44 | 64 | 38 | 146 | 15 | 33 | 13 | 61 |
| GMT (95% CI) | 8.0 NE, NE | 66.2 (37.7, 116.2) | 139.4 (92.6, 209.7) | 156.0 (88.7, 274.2) | 114.7 (86.1, 152.7) | 5686.3 (1679.5, 19252.2) | 11371.6 (7988.2, 16188.2) | 20364.9 (13679.1, 30318.5) | 10857.7 (7600.8, 15510.2) |
| GMFR (95% CI) | 1.00 NE, NE | 8.27 (4.71, 14.52) | 17.42 (11.58, 26.22) | 19.50 (11.09, 34.28) | 14.33 (10.76, 19.08) | 2.27 (1.40, 3.66) | 2.42 (1.80, 3.25) | 2.87 (1.76, 4.67) | 2.47 (2.00, 3.05) |
| **Month 2** | | | | | | | | | |
| *n* | 41 | 38 | 48 | 35 | 121 | 15 | 25 | 11 | 51 |
| GMT (95% CI) | 8.0 NE, NE | 33.8 (19.8, 57.7) | 75.3 (43.7, 129.5) | 84.2 (51.2, 138.5) | 60.5 (44.4, 82.3) | 5448.5 (1547.6, 19181.8) | 9808.0 (6008.7, 16009.6) | 11983.0 (7109.1, 20198.5) | 8615.0 (5603.4, 13245.1) |
| GMFR (95% CI) | 1.00 NE, NE | 4.22 (2.48, 7.21) | 9.41 (5.47, 16.19) | 10.53 (6.40, 17.32) | 7.56 (5.55, 10.29) | 2.17 (1.43, 3.29) | 2.13 (1.59, 2.86) | 1.68 (1.04, 2.73) | 2.04 (1.66, 2.49) |
| **Month 3** | | | | | | | | | |
| *n* | 46 | 27 | 56 | 36 | 119 | 15 | 29 | 13 | 57 |

| Timepoint | CMV-Seronegative Placebo (N=53) | CMV-Seronegative mRNA-1647 50 µg (N=44) | CMV-Seronegative mRNA-1647 100 µg (N=64) | CMV-Seronegative mRNA-1647 150 µg (N=44) | CMV-Seronegative mRNA-1647 Total (N=152) | CMV-Seropositive mRNA-1647 50 µg (N=15) | CMV-Seropositive mRNA-1647 100 µg (N=34) | CMV-Seropositive mRNA-1647 150 µg (N=15) | CMV-Seropositive mRNA-1647 Total (N=64) |
|---|---|---|---|---|---|---|---|---|---|
| GMT (95% CI) | 8.0 NE, NE | 4395.3 (3194.6, 6047.3) | 3981.2 (3126.7, 5069.2) | 4596.5 (3437.6, 6146.1) | 4252.5 (3635.6, 4974.1) | 10410.2 (6923.1, 15653.7) | 10130.0 (7609.8, 13484.8) | 12868.2 (9234.1, 17932.5) | 10775.2 (8944.2, 12981.0) |
| GMFR (95% CI) | 1.00 NE, NE | 549.41 (399.33, 755.91) | 497.65 (390.84, 633.65) | 574.56 (429.70, 768.26) | 531.57 (454.45, 621.77) | 4.15 (1.33, 12.94) | 2.23 (1.52, 3.27) | 2.06 (1.29, 3.27) | 2.58 (1.81, 3.67) |
| **Month 6** | | | | | | | | | |
| *n* | 32 | 32 | 36 | 28 | 96 | 14 | 19 | 10 | 43 |
| GMT (95% CI) | 9.0 (7.1, 11.5) | 346.4 (219.5, 546.6) | 562.4 (353.6, 894.7) | 517.8 (354.4, 756.5) | 467.1 (363.4, 600.3) | 7447.4 (3921.5, 14143.7) | 10185.5 (6627.2, 15654.3) | 16061.7 (11656.7, 22131.2) | 10226.2 (7702.8, 13576.2) |
| GMFR (95% CI) | 1.13 (0.88, 1.44) | 43.29 (27.44, 68.32) | 70.30 (44.19, 111.83) | 64.73 (44.31, 94.57) | 58.39 (45.43, 75.04) | 3.37 (0.96, 11.80) | 1.65 (1.28, 2.12) | 2.22 (1.10, 4.46) | 2.23 (1.46, 3.40) |
| **Month 7** | | | | | | | | | |
| *n* | 37 | 27 | 40 | 27 | 94 | 13 | 24 | 11 | 48 |
| GMT (95% CI) | 8.0 NE, NE | 2758.2 (1957.6, 3886.3) | 3811.4 (2962.7, 4903.3) | 3761.2 (2849.3, 4965.0) | 3460.1 (2941.4, 4070.3) | 9013.2 (5366.4, 15138.0) | 9170.7 (6588.8, 12764.2) | 13122.9 (9167.8, 18784.3) | 9909.0 (7954.2, 12344.1) |

| Timepoint | CMV-Seronegative | | | | | CMV-Seropositive | | | |
|---|---|---|---|---|---|---|---|---|---|
| | | **mRNA-1647** | | | | **mRNA-1647** | | | |
| | Placebo (N=53) | 50 µg (N=44) | 100 µg (N=64) | 150 µg (N=44) | Total (N=152) | 50 µg (N=15) | 100 µg (N=34) | 150 µg (N=15) | Total (N=64) |
| GMFR (95% CI) | 1.00 NE, NE | 344.78 (244.70, 485.79) | 476.43 (370.34, 612.91) | 470.15 (356.16, 620.63) | 432.51 (367.67, 508.79) | 4.20 (1.06, 16.74) | 1.67 (1.21, 2.32) | 2.02 (1.07, 3.82) | 2.24 (1.49, 3.37) |
| **Month 12** | | | | | | | | | |
| *n* | 34 | 27 | 39 | 27 | 93 | 14 | 18 | 12 | 44 |
| GMT (95% CI) | 9.8 (6.5, 14.8) | 1065.0 (664.7, 1706.2) | 1168.7 (807.0, 1692.3) | 1227.0 (829.7, 1814.8) | 1153.8 (919.1, 1448.4) | 8442.3 (5216.3, 13663.4) | 8793.6 (5463.8, 14152.7) | 11657.2 (7701.7, 17644.2) | 9373.9 (7269.0, 12088.3) |
| GMFR (95% CI) | 1.23 (0.81, 1.85) | 133.12 (83.09, 213.28) | 146.08 (100.88, 211.54) | 153.38 (103.71, 226.85) | 144.22 (114.88, 181.05) | 3.82 (1.13, 12.87) | 1.53 (1.20, 1.94) | 1.80 (0.84, 3.84) | 2.14 (1.40, 3.27) |
| **Month 18** | | | | | | | | | |
| *n* | 36 | 22 | 36 | 23 | 81 | 14 | 17 | 11 | 42 |
| GMT (95% CI) | 11.6 (7.1, 19.0) | 486.3 (234.5, 1008.7) | 582.3 (355.1, 955.1) | 817.1 (526.6, 1267.8) | 610.5 (447.6, 832.6) | 7023.2 (4003.5, 12320.5) | 7447.5 (4216.6, 13153.9) | 9860.9 (6933.7, 14023.9) | 7860.4 (5883.2, 10502.2) |
| GMFR (95% CI) | 1.45 (0.89, 2.38) | 60.79 (29.31, 126.09) | 72.79 (44.38, 119.38) | 102.13 (65.82, 158.48) | 76.31 (55.95, 104.08) | 3.17 (0.97, 10.41) | 1.29 (1.03, 1.61) | 1.39 (0.72, 2.65) | 1.77 (1.17, 2.70) |

| Timepoint | CMV-Seronegative | | | | | CMV-Seropositive | | | |
|---|---|---|---|---|---|---|---|---|---|
| | | mRNA-1647 | | | | mRNA-1647 | | | |
| | Placebo (N=53) | 50 µg (N=44) | 100 µg (N=64) | 150 µg (N=44) | Total (N=152) | 50 µg (N=15) | 100 µg (N=34) | 150 µg (N=15) | Total (N=64) |
| **Anti-gB Binding Antibodies[c]** | | | | | | | | | |
| **Baseline** | | | | | | | | | |
| *n* | 53 | 44 | 64 | 44 | 152 | 15 | 34 | 15 | 64 |
| GMT (95% CI) | 17.50 NE, NE | 17.50 NE, NE | 18.20 (16.83, 19.68) | 17.50 NE, NE | 17.79 (17.22, 18.38) | 1146.61 (367.95, 3573.08) | 2631.32 (1611.38, 4296.84) | 2981.12 (1649.35, 5388.25) | 2230.11 (1518.78, 3274.58) |
| **Month 1** | | | | | | | | | |
| *n* | 51 | 44 | 64 | 38 | 146 | 15 | 33 | 13 | 61 |
| GMT (95% CI) | 17.50 NE, NE | 19.05 (17.53, 20.70) | 21.73 (18.85, 25.06) | 19.65 (17.79, 21.71) | 20.35 (18.94, 21.85) | 2121.17 (645.07, 6974.93) | 4849.42 (2999.05, 7841.45) | 6754.86 (3799.57, 12008.74) | 4246.74 (2852.16, 6323.21) |
| GMFR (95% CI) | 1.000 NE, NE | 1.088 (1.002, 1.183) | 1.194 (1.044, 1.365) | 1.123 (1.016, 1.241) | 1.143 (1.068, 1.223) | 1.850 (1.283, 2.666) | 1.873 (1.522, 2.305) | 2.128 (1.529, 2.962) | 1.919 (1.650, 2.231) |
| **Month 2** | | | | | | | | | |
| *n* | 41 | 38 | 48 | 35 | 121 | 15 | 25 | 11 | 51 |
| GMT (95% CI) | 19.95 (15.31, 25.98) | 17.82 (17.18, 18.49) | 24.44 (19.20, 31.12) | 18.53 (17.06, 20.12) | 20.43 (18.47, 22.59) | 1998.18 (627.93, 6358.51) | 4383.36 (2308.90, 8321.64) | 5908.63 (3097.70, 11270.27) | 3710.50 (2328.94, 5911.61) |
| GMFR (95% CI) | 1.140 (0.875, 1.485) | 1.018 (0.981, 1.057) | 1.326 (1.089, 1.615) | 1.059 (0.975, 1.150) | 1.144 (1.052, 1.243) | 1.743 (1.243, 2.443) | 1.746 (1.449, 2.104) | 1.954 (1.349, 2.831) | 1.788 (1.547, 2.067) |
| **Month 3** | | | | | | | | | |

| Timepoint | CMV-Seronegative | | | | | CMV-Seropositive | | | |
|---|---|---|---|---|---|---|---|---|---|
| | | mRNA-1647 | | | | mRNA-1647 | | | |
| | Placebo (N=53) | 50 µg (N=44) | 100 µg (N=64) | 150 µg (N=44) | Total (N=152) | 50 µg (N=15) | 100 µg (N=34) | 150 µg (N=15) | Total (N=64) |
| *n* | 46 | 27 | 56 | 36 | 119 | 15 | 29 | 13 | 57 |
| GMT (95% CI) | 17.50 NE, NE | 339.83 (234.71, 492.04) | 480.55 (360.83, 639.99) | 347.45 (268.76, 449.18) | 402.71 (338.18, 479.54) | 3699.02 (2149.36, 6365.94) | 4708.14 (3215.43, 6893.83) | 8112.05 (5108.43, 12881.71) | 5002.29 (3862.97, 6477.63) |
| GMFR (95% CI) | 1.000 NE, NE | 19.419 (13.412, 28.116) | 26.259 (19.197, 35.919) | 19.854 (15.357, 25.668) | 22.533 (18.754, 27.073) | 3.226 (1.552, 6.705) | 1.969 (1.495, 2.593) | 2.863 (1.877, 4.368) | 2.442 (1.911, 3.120) |
| **Month 6** | | | | | | | | | |
| *n* | 32 | 32 | 36 | 28 | 96 | 14 | 19 | 10 | 43 |
| GMT (95% CI) | 18.63 (16.40, 21.17) | 83.84 (57.61, 122.00) | 133.80 (90.44, 197.94) | 107.28 (75.34, 152.76) | 107.35 (86.61, 133.05) | 3384.91 (1634.19, 7011.21) | 5137.05 (3042.33, 8674.05) | 10702.55 (6307.14, 18161.07) | 5319.40 (3744.81, 7556.07) |
| GMFR (95% CI) | 1.065 (0.937, 1.210) | 4.791 (3.292, 6.971) | 7.132 (4.867, 10.451) | 6.130 (4.305, 8.729) | 5.976 (4.838, 7.383) | 3.397 (1.441, 8.012) | 1.535 (1.225, 1.924) | 3.408 (1.578, 7.359) | 2.393 (1.712, 3.346) |
| **Month 7** | | | | | | | | | |
| *n* | 37 | 27 | 40 | 27 | 94 | 13 | 24 | 11 | 48 |
| GMT (95% CI) | 17.50 NE, NE | 1154.82 (848.30, 1572.11) | 962.89 (680.53, 1362.42) | 937.17 (686.85, 1278.70) | 1006.64 (833.96, 1215.07) | 4143.99 (2473.48, 6942.73) | 4971.82 (3291.99, 7508.85) | 10115.96 (5983.30, 17103.06) | 5569.16 (4230.24, 7331.86) |
| GMFR (95% CI) | 1.000 NE, NE | 65.990 | 51.684 | 53.552 | 56.010 | 4.210 | 1.746 (1.392, 2.192) | 3.466 (1.748, 6.869) | 2.593 (1.867, 3.603) |

| Timepoint | CMV-Seronegative Placebo (N=53) | CMV-Seronegative mRNA-1647 50 µg (N=44) | CMV-Seronegative mRNA-1647 100 µg (N=64) | CMV-Seronegative mRNA-1647 150 µg (N=44) | CMV-Seronegative mRNA-1647 Total (N=152) | CMV-Seropositive mRNA-1647 50 µg (N=15) | CMV-Seropositive mRNA-1647 100 µg (N=34) | CMV-Seropositive mRNA-1647 150 µg (N=15) | CMV-Seropositive mRNA-1647 Total (N=64) |
|---|---|---|---|---|---|---|---|---|---|
| | | (48.474, 89.835) | (33.457, 79.840) | (39.249, 73.069) | (45.056, 69.628) | (1.489, 11.904) | | | |
| **Month 12** | | | | | | | | | |
| *n* | 34 | 27 | 39 | 27 | 93 | 14 | 18 | 12 | 44 |
| GMT (95% CI) | 20.52 (14.85, 28.35) | 296.60 (197.73, 444.92) | 396.96 (269.50, 584.72) | 261.98 (195.16, 351.69) | 323.30 (261.11, 400.30) | 3746.85 (1860.42, 7546.09) | 4130.37 (2426.85, 7029.68) | 8915.36 (5714.18, 13909.90) | 4939.18 (3561.20, 6850.36) |
| GMFR (95% CI) | 1.172 (0.848, 1.620) | 16.949 (11.299, 25.424) | 21.273 (13.981, 32.368) | 14.971 (11.152, 20.097) | 17.984 (14.394, 22.469) | 3.761 (1.475, 9.589) | 1.308 (0.704, 2.431) | 3.009 (1.740, 5.205) | 2.297 (1.523, 3.467) |
| **Month 18** | | | | | | | | | |
| *n* | 36 | 22 | 36 | 23 | 81 | 14 | 17 | 11 | 42 |
| GMT (95% CI) | 20.62 (14.78, 28.79) | 170.25 (103.18, 280.91) | 225.31 (128.30, 395.70) | 156.47 (107.51, 227.73) | 188.26 (140.15, 252.89) | 2754.18 (1311.43, 5784.14) | 3037.74 (1743.56, 5292.56) | 7665.63 (5045.24, 11646.99) | 3746.71 (2640.32, 5316.70) |
| GMFR (95% CI) | 1.179 (0.844, 1.645) | 9.728 (5.896, 16.052) | 12.875 (7.331, 22.611) | 8.941 (6.144, 13.013) | 10.758 (8.009, 14.451) | 2.764 (1.171, 6.529) | 1.006 (0.529, 1.914) | 2.535 (1.370, 4.691) | 1.795 (1.188, 2.712) |
| **Anti-Pentamer Binding Antibodies[d]** | | | | | | | | | |
| **Baseline** | | | | | | | | | |
| *n* | 53 | 44 | 64 | 44 | 152 | 15 | 34 | 15 | 64 |
| GMT | 24.0 | 24.0 | 24.0 | 24.0 | 24.0 | 334.7 | 412.1 | 496.3 | 410.0 |

| Timepoint | CMV-Seronegative Placebo (N=53) | CMV-Seronegative mRNA-1647 50 µg (N=44) | CMV-Seronegative mRNA-1647 100 µg (N=64) | CMV-Seronegative mRNA-1647 150 µg (N=44) | CMV-Seronegative mRNA-1647 Total (N=152) | CMV-Seropositive mRNA-1647 50 µg (N=15) | CMV-Seropositive mRNA-1647 100 µg (N=34) | CMV-Seropositive mRNA-1647 150 µg (N=15) | CMV-Seropositive mRNA-1647 Total (N=64) |
|---|---|---|---|---|---|---|---|---|---|
| (95% CI) | NE, NE | NE, NE | NE, NE | NE, NE | NE, NE | (143.4, 781.2) | (283.7, 598.7) | (273.1, 902.2) | (305.1, 550.8) |
| **Month 1** | | | | | | | | | |
| *n* | 51 | 44 | 64 | 38 | 146 | 15 | 33 | 13 | 61 |
| GMT (95% CI) | 24.5 (23.5, 25.6) | 165.8 (113.1, 243.1) | 148.9 (105.7, 209.6) | 518.7 (381.3, 705.7) | 212.8 (171.0, 264.9) | 1905.2 (836.5, 4339.2) | 4114.8 (2790.1, 6068.5) | 4655.2 (3036.5, 7136.8) | 3495.7 (2587.4, 4723.0) |
| GMFR (95% CI) | 1.02 (0.98, 1.07) | 6.91 (4.71, 10.13) | 6.20 (4.41, 8.73) | 21.61 (15.89, 29.40) | 8.87 (7.12, 11.04) | 5.69 (3.32, 9.77) | 10.00 (6.66, 15.00) | 8.01 (4.19, 15.34) | 8.30 (6.27, 11.00) |
| **Month 2** | | | | | | | | | |
| *n* | 41 | 38 | 48 | 35 | 121 | 15 | 25 | 11 | 51 |
| GMT (95% CI) | 24.0 NE, NE | 110.1 (75.3, 161.0) | 112.1 (76.2, 164.9) | 278.3 (202.4, 382.5) | 145.0 (116.1, 181.0) | 1371.3 (627.0, 2998.9) | 3017.4 (1699.1, 5358.6) | 3476.4 (2259.2, 5349.4) | 2466.9 (1705.9, 3567.5) |
| GMFR (95% CI) | 1.00 NE, NE | 4.59 (3.14, 6.71) | 4.67 (3.17, 6.87) | 11.59 (8.43, 15.94) | 6.04 (4.84, 7.54) | 4.10 (2.51, 6.69) | 7.41 (5.02, 10.93) | 6.60 (3.33, 13.07) | 6.07 (4.63, 7.95) |
| **Month 3** | | | | | | | | | |
| *n* | 46 | 27 | 56 | 36 | 119 | 15 | 29 | 13 | 57 |
| GMT (95% CI) | 24.0 NE, NE | 5750.0 (4006.7, 8251.8) | 3165.2 (2112.9, 4741.7) | 5841.8 (4481.9, 7614.3) | 4362.6 (3487.8, 5456.6) | 3378.1 (2377.3, 4800.3) | 4159.7 (2770.0, 6246.7) | 4556.1 (3063.5, 6775.9) | 4020.6 (3182.2, 5079.9) |
| GMFR | 1.00 | 239.58 | 131.88 | 243.41 | 181.77 | 10.09 | 9.82 | 10.21 | 9.98 |

| | CMV-Seronegative | | | | | CMV-Seropositive | | | |
|---|---|---|---|---|---|---|---|---|---|
| | | mRNA-1647 | | | | mRNA-1647 | | | |
| Timepoint | Placebo (N=53) | 50 µg (N=44) | 100 µg (N=64) | 150 µg (N=44) | Total (N=152) | 50 µg (N=15) | 100 µg (N=34) | 150 µg (N=15) | Total (N=64) |
| (95% CI) | NE, NE | (166.94, 343.82) | (88.04, 197.57) | (186.74, 317.26) | (145.33, 227.36) | (4.21, 24.20) | (6.96, 13.85) | (5.09, 20.46) | (7.36, 13.53) |
| **Month 6** | | | | | | | | | |
| *n* | 32 | 32 | 36 | 28 | 96 | 14 | 19 | 10 | 43 |
| GMT (95% CI) | 26.2 (21.9, 31.5) | 573.1 (387.6, 847.3) | 597.0 (393.9, 905.0) | 661.6 (468.8, 933.6) | 606.9 (487.5, 755.4) | 2139.1 (1240.2, 3689.7) | 2447.0 (1485.4, 4031.0) | 2831.4 (1932.1, 4149.3) | 2423.0 (1837.5, 3195.1) |
| GMFR (95% CI) | 1.09 (0.91, 1.31) | 23.88 (16.15, 35.31) | 24.88 (16.41, 37.71) | 27.56 (19.53, 38.90) | 25.29 (20.31, 31.48) | 6.24 (2.46, 15.79) | 4.84 (3.28, 7.15) | 5.39 (2.32, 12.54) | 5.39 (3.75, 7.75) |
| **Month 7** | | | | | | | | | |
| *n* | 37 | 27 | 40 | 27 | 94 | 13 | 24 | 11 | 48 |
| GMT (95% CI) | 24.0 NE, NE | 5572.6 (3871.2, 8021.9) | 3709.7 (2273.7, 6052.5) | 6512.6 (4542.7, 9336.7) | 4901.2 (3809.7, 6305.5) | 3349.3 (2009.3, 5582.8) | 2891.7 (2014.5, 4150.8) | 3239.1 (2121.4, 4945.8) | 3088.3 (2452.5, 3888.9) |
| GMFR (95% CI) | 1.00 NE, NE | 232.19 (161.30, 334.25) | 154.57 (94.74, 252.19) | 271.36 (189.28, 389.03) | 204.22 (158.74, 262.73) | 8.81 (3.09, 25.09) | 6.30 (4.50, 8.81) | 6.84 (3.66, 12.79) | 7.03 (5.06, 9.76) |
| **Month 12** | | | | | | | | | |
| *n* | 34 | 27 | 39 | 27 | 93 | 14 | 18 | 12 | 44 |
| GMT (95% CI) | 28.0 (20.5, 38.2) | 1230.0 (810.2, 1867.5) | 1623.5 (1127.0, 2338.7) | 1458.9 (943.4, 2255.9) | 1452.0 (1159.3, 1818.7) | 1629.3 (993.9, 2670.8) | 2314.9 (1364.4, 3927.3) | 2177.7 (1715.9, 2763.9) | 2035.9 (1570.6, 2639.1) |

| Timepoint | CMV-Seronegative Placebo (N=53) | CMV-Seronegative mRNA-1647 50 µg (N=44) | CMV-Seronegative mRNA-1647 100 µg (N=64) | CMV-Seronegative mRNA-1647 150 µg (N=44) | CMV-Seronegative mRNA-1647 Total (N=152) | CMV-Seropositive mRNA-1647 50 µg (N=15) | CMV-Seropositive mRNA-1647 100 µg (N=34) | CMV-Seropositive mRNA-1647 150 µg (N=15) | CMV-Seropositive mRNA-1647 Total (N=64) |
|---|---|---|---|---|---|---|---|---|---|
| GMFR (95% CI) | 1.17 (0.85, 1.59) | 51.25 (33.76, 77.81) | 67.64 (46.96, 97.45) | 60.79 (39.31, 93.99) | 60.50 (48.30, 75.78) | 4.75 (2.05, 11.01) | 4.97 (2.84, 8.69) | 4.43 (2.39, 8.23) | 4.75 (3.32, 6.79) |
| **Month 18** | | | | | | | | | |
| *n* | 36 | 22 | 36 | 23 | 81 | 14 | 17 | 11 | 42 |
| GMT (95% CI) | 27.1 (21.1, 34.8) | 508.9 (313.1, 827.2) | 815.9 (553.1, 1203.6) | 796.5 (496.9, 1276.6) | 712.8 (556.0, 913.9) | 1020.9 (618.6, 1684.8) | 1361.5 (919.0, 2017.0) | 1644.4 (1232.2, 2194.4) | 1299.6 (1032.0, 1636.6) |
| GMFR (95% CI) | 1.13 (0.88, 1.45) | 21.20 (13.04, 34.47) | 34.00 (23.05, 50.15) | 33.19 (20.70, 53.19) | 29.70 (23.17, 38.08) | 2.98 (1.47, 6.02) | 2.86 (1.83, 4.48) | 3.12 (1.59, 6.12) | 2.97 (2.17, 4.06) |

Abbreviations: CI, confidence interval; GMT, geometric mean titer; GMFR, geometric mean fold-rise (change in GMT relative to baseline [Month 0, before administration of study vaccine]); LLOQ, lower limit of quantification; ULOQ, upper limit of quantification.

[a]Neutralizing antibodies epithelial cell: LLOQ = 16; ULOQ=33,554,432.

[b]Neutralizing antibodies against fibroblast: LLOQ=16; ULOQ=16,777,216.

[c]Anti-gB antibodies in fold dilution: LLOQ=35; ULOQ: 991,287.

[d]Anti-Pentamer antibodies in fold dilution: LLOQ=48; ULOQ=117,612.

N is the number of participants in the per-protocol set for antibody-mediated immunogenicity (Part 1 and Part 2 combined).

*n* is the number of participants with non-missing data at baseline or at the corresponding timepoint.

95% CIs were calculated based on the t-distribution of the log-transformed values or the difference in the log-transformed values for GMT and GMR, respectively, then back-transformed to the original scale for presentation.

**Table S5. Neutralizing and binding antibody titers after vaccination with mRNA-1647 by CMV serostatus through Month 54 (Extension Trial; per-protocol set for antibody-mediated immunogenicity)**

| | CMV-Seronegative | | | | | CMV-Seropositive | | | |
|---|---|---|---|---|---|---|---|---|---|
| | | mRNA-1647 | | | | mRNA-1647 | | | |
| Timepoint | Placebo (N=22) | 50 µg (N=14) | 100 µg (N=24) | 150 µg (N=18) | Total (N=56) | 50 µg (N=11) | 100 µg (N=12) | 150 µg (N=9) | Total (N=32) |
| **Neutralizing Antibodies Against Epithelial Cell Infection[a]** | | | | | | | | | |
| **Baseline** | | | | | | | | | |
| *n* | 22 | 14 | 24 | 18 | 56 | 11 | 12 | 9 | 32 |
| GMT (95% CI) | 8.00 NE, NE | 8.00 NE, NE | 8.00 NE, NE | 8.00 NE, NE | 8.00 NE, NE | 1553.4 (248.5-9709.9) | 5043.3 (2739.8-9283.7) | 8596.6 (3609.9-20472.2) | 3908.8 (1978.9-7720.9) |
| **Month 1** | | | | | | | | | |
| *n* | 22 | 14 | 24 | 15 | 53 | 11 | 12 | 8 | 31 |
| GMT (95% CI) | 8.00 NE, NE | 843.9 (292.5-2434.4) | 1048.9 (389.0-2828.3) | 2987.9 (1600.7-5577.2) | 1331.8 (773.0- 2294.6) | 22846.1 (3650.5-142977.9) | 73051.1 (30443.1-175293.3) | 120085.1 (46151.9-312455.8) | 54980.1 (26490.1-114110.9) |
| GMFR (95% CI) | 1.00 NE, NE | 105.48 (36.57-304.30) | 131.11 (48.63-353.54) | 373.48 (200.09-697.15) | 166.48 (96.63-286.82) | 14.71 (8.69-24.89) | 14.48 (5.61-37.38) | 14.25 (3.51-57.87) | 14.50 (9.02-23.32) |
| **Month 2** | | | | | | | | | |
| *n* | 22 | 14 | 24 | 18 | 56 | 11 | 12 | 9 | 32 |
| GMT 95% CI | 8.00 NE, NE | 882.5 (370.0-2105.0) | 581.4 (254.5-1327.8) | 1596.8 (923.5-2760.8) | 892.9 (575.0- 1386.6) | 23063.8 (3576.6-148727.5) | 49978.4 (25633.0-97446.2) | 79972.0 (39627.6-161390.8) | 43727.3 (22607.1-84578.4) |
| GMFR (95% CI) | 1.00 NE, NE | 110.31 (46.24-263.12) | 72.67 (31.82-165.97) | 199.59 (115.44-345.10) | 111.61 (71.87-173.33) | 14.85 (7.39-29.82) | 9.91 (4.03-24.37) | 9.30 (4.03-21.46) | 11.19 (7.29-17.16) |
| **Month 3** | | | | | | | | | |

| | CMV-Seronegative | | | | | CMV-Seropositive | | | |
|---|---|---|---|---|---|---|---|---|---|
| | | mRNA-1647 | | | | mRNA-1647 | | | |
| Timepoint | Placebo (N=22) | 50 µg (N=14) | 100 µg (N=24) | 150 µg (N=18) | Total (N=56) | 50 µg (N=11) | 100 µg (N=12) | 150 µg (N=9) | Total (N=32) |
| *n* | 21 | 9 | 24 | 18 | 51 | 11 | 12 | 9 | 32 |
| GMT (95% CI) | 8.00 NE, NE | 94544.3 (44381.9-201402.4) | 60925.8 (31927.5-116261.8) | 50570.6 (30558.7-83687.7) | 61648.7 (43059.5-88262.9) | 92286.4 (56934.9-149587.8) | 84043.1 (43500.1-162373.0) | 120680.5 (65169.9-223473.9) | 96087.0 (70518.2-130926.8) |
| GMFR (95% CI) | 1.00 NE, NE | 11818.04 (5547.74-25175.30) | 7615.72 (3990.94-14532.72) | 6321.33 (3819.84-10460.97) | 7706.08 (5382.44-11032.86) | 59.41 (10.43-338.49) | 16.66 (7.31-37.98) | 14.04 (6.78-29.05) | 24.58 (12.62-47.87) |
| **Month 6** | | | | | | | | | |
| *n* | 22 | 14 | 24 | 18 | 56 | 11 | 12 | 9 | 32 |
| GMT (95% CI) | 8.00 NE, NE | 10396.7 (6147.6-17582.5) | 10081.6 (5462.9-18605.3) | 9375.4 (6237.3-14092.4) | 9925.1 (7325.9-13446.3) | 34994.5 (21520.5-56904.6) | 62887.9 (30301.1-130519.5) | 69990.2 (39272.6-124733.9) | 52982.1 (37790.7-74280.3) |
| GMFR (95% CI) | 1.00 NE, NE | 1299.58 (768.45-2197.82) | 1260.20 (682.86-2325.67) | 1171.93 (779.66-1761.56) | 1240.63 (915.74-1680.79) | 22.53 (4.32-117.35) | 12.47 (5.19-29.93) | 8.14 (3.85-17.23) | 13.55 (7.23-25.40) |
| **Month 7** | | | | | | | | | |
| *n* | 22 | 13 | 23 | 18 | 54 | 11 | 12 | 9 | 32 |
| GMT (95% CI) | 8.00 NE, NE | 108214.9 (47856.5-244699.5) | 102870.2 (61593.1-171809.5) | 93047.5 (63009.6-137405.2) | 100706.4 (74703.7-135760.1) | 73470.3 (43470.6-124173.2) | 90371.0 (40225.8-203027.1) | 99955.3 (49850.7-200419.4) | 86582.9 (60370.3-124176.9) |
| GMFR (95% CI) | 1.00 NE, NE | 13526.86 (5982.06-30587.44) | 12858.77 (7699.14-21476.18) | 11630.94 (7876.20-17175.65) | 12588.30 (9337.96-16970.01) | 47.30 (6.22-359.75) | 17.92 (7.03-45.68) | 11.63 (5.92-22.82) | 22.15 (10.53-46.59) |
| **Month 12** | | | | | | | | | |
| *n* | 20 | 14 | 23 | 18 | 55 | 11 | 12 | 9 | 32 |
| GMT (95% CI) | 8.00 NE, NE | 23118.9 | 13888.0 | 22788.5 | 18593.7 | 31789.5 | 25980.0 | 56145.2 | 34585.5 |

| Timepoint | CMV-Seronegative | | | | | CMV-Seropositive | | | |
|---|---|---|---|---|---|---|---|---|---|
| | | mRNA-1647 | | | | mRNA-1647 | | | |
| | Placebo (N=22) | 50 µg (N=14) | 100 µg (N=24) | 150 µg (N=18) | Total (N=56) | 50 µg (N=11) | 100 µg (N=12) | 150 µg (N=9) | Total (N=32) |
| | | (11620.3-45995.5) | (9051.4-21309.1) | (12876.8-40329.6) | (13796.9-25058.2) | (19104.6-52896.8) | (15181.0-44461.1) | (27568.1-114345.2) | (25245.0-47381.9) |
| GMFR (95% CI) | 1.00 NE, NE | 2889.86 (1452.54-5749.43) | 1736.00 (1131.42-2663.64) | 2848.56 (1609.60-5041.20) | 2324.21 (1724.62-3132.27) | 20.46 (3.73-112.23) | 5.15 (2.19-12.13) | 6.53 (2.67-15.99) | 8.85 (4.53-17.29) |
| **Month 18** | | | | | | | | | |
| *n* | 21 | 14 | 24 | 18 | 56 | 11 | 12 | 9 | 32 |
| GMT (95% CI) | 8.5 (7.5-9.6) | 12505.5 (6717.4-23280.8) | 8441.6 (5510.7-12931.6) | 12768.1 (7228.5-22553.0) | 10637.9 (7986.5-14169.5) | 21072.2 (12321.8-36036.8) | 19137.7 (12597.9-29072.3) | 36162.3 (16165.6-80895.1) | 23658.8 (17434.4-32105.6) |
| GMFR (95% CI) | 1.06 (0.94-1.20) | 1563.19 (839.68-2910.10) | 1055.21 (688.83-1616.45) | 1596.02 (903.57-2819.13) | 1329.74 (998.31-1771.19) | 13.57 (2.78-66.27) | 3.79 (1.80-8.00) | 4.21 (1.78-9.95) | 6.05 (3.25-11.26) |
| **Month 24** | | | | | | | | | |
| *n* | 20 | 13 | 23 | 18 | 54 | 11 | 12 | 9 | 32 |
| GMT (95% CI) | 8.4 (7.6-9.1) | 12634.9 (6337.9-25188.3) | 5776.4 (2610.6-12781.3) | 11285.0 (7744.6-16443.9) | 8718.4 (5921.3-12836.8) | 21388.9 (13083.9-34965.4) | 20457.7 (14211.4-29449.5) | 26961.3 (19017.2-38223.9) | 22450.2 (18102.4-27842.1) |
| GMFR (95% CI) | 1.04 (0.95-1.14) | 1579.37 (792.24-3148.54) | 722.05 (326.32-1597.66) | 1410.63 (968.07-2055.49) | 1089.80 (740.17-1604.59) | 13.77 (2.71-70.06) | 4.06 (1.86-8.87) | 3.14 (1.53-6.43) | 5.74 (3.05-10.81) |
| **Month 30** | | | | | | | | | |
| *n* | 21 | 11 | 21 | 17 | 49 | 11 | 12 | 8 | 31 |
| GMT (95% CI) | 11.8 (5.8-23.9) | 8144.3 (3969.2-16711.3) | 10270.4 (6425.0-16417.4) | 10575.8 (6583.4-16989.4) | 9848.9 (7416.7-13078.9) | 16993.5 (9939.2-29054.5) | 13938.6 (9188.2-21145.1) | 28614.9 (16005.0-51159.8) | 18004.1 (13657.0-23734.7) |
| GMFR | 1.48 | 1018.04 | 1283.80 | 1321.98 | 1231.12 | 10.94 | 2.76 | 3.12 | 4.65 |

| | CMV-Seronegative | | | | | CMV-Seropositive | | | |
|---|---|---|---|---|---|---|---|---|---|
| | | mRNA-1647 | | | | mRNA-1647 | | | |
| Timepoint | Placebo (N=22) | 50 µg (N=14) | 100 µg (N=24) | 150 µg (N=18) | Total (N=56) | 50 µg (N=11) | 100 µg (N=12) | 150 µg (N=9) | Total (N=32) |
| (95% CI) | (0.73-2.98) | (496.15-2088.92) | (803.12-2052.18) | (822.93-2123.67) | (927.08-1634.86) | (2.17-55.24) | (1.29-5.93) | (1.31-7.43) | (2.43-8.90) |
| **Month 36** | | | | | | | | | |
| *n* | 21 | 11 | 22 | 16 | 49 | 10 | 11 | 8 | 29 |
| GMT (95% CI) | 8.00 NE, NE | 7538.9 (3575.9-15894.2) | 7710.4 (5155.3-11531.8) | 8177.2 (5661.6-11810.4) | 7820.2 (6080.7-10057.4) | 15043.0 (8828.4-25632.4) | 19329.2 (13711.2-27249.0) | 19013.8 (14402.9-25100.8) | 17648.1 (14263.9-21835.2) |
| GMFR (95% CI) | 1.00 NE, NE | 942.36 (446.98-1986.77) | 963.80 (644.42-1441.47) | 1022.15 (707.70-1476.30) | 977.53 (760.09-1257.17) | 11.71 (1.91-71.61) | 3.81 (1.87-7.74) | 2.08 (0.87-4.97) | 4.74 (2.39-9.40) |
| Month 42 | | | | | | | | | |
| *n* | 18 | 9 | 18 | 13 | 40 | 9 | 10 | 8 | 27 |
| GMT (95% CI) | 11.9 (5.1- 27.6) | 7120.8 (3612.8-14035.0) | 5915.3 (3407.4-10269.3) | 10548.2 (6307.1-17641.3) | 7442.9 (5419.3-10222.3) | 14930.1 (7882.8-28278.0) | 13530.9 (9306.6-19672.6) | 19271.3 (10513.0-35326.0) | 15526.7 (11835.7-20368.7) |
| GMFR (95% CI) | 1.49 (0.64- 3.45) | 890.10 (451.60-1754.37) | 739.42 (425.92-1283.66) | 1318.53 (788.38-2205.16) | 930.36 (677.41-1277.78) | 6.61 (1.24- 35.22) | 2.71 (1.33- 5.53) | 2.10 (0.78- 5.69) | 3.38 (1.83- 6.27) |
| Month 48 | | | | | | | | | |
| *n* | 17 | 9 | 17 | 14 | 40 | 9 | 10 | 8 | 27 |
| GMT (95% CI) | 8.0 NE, NE | 6744.9 (3533.9-12873.5) | 5023.1 (2682.9-9404.5) | 7320.8 (4698.0-11407.9) | 6123.9 (4459.9-8408.7) | 14755.0 (9554.3-22786.4) | 19337.1 (12887.5-29014.3) | 15206.3 (11819.9-19563.0) | 16455.7 (13501.4-20056.5) |
| GMFR (95% CI) | 1.00 NE, NE | 843.11 (441.73-1609.19) | 627.89 (335.36-1175.57) | 915.10 (587.25-1425.98) | 765.49 (557.49-1051.09) | 6.53 (1.58- 27.04) | 3.87 (1.76- 8.53) | 1.66 (0.70- 3.93) | 3.59 (2.03- 6.33) |
| Month 54 | | | | | | | | | |

| Timepoint | CMV-Seronegative | | | | | CMV-Seropositive | | | |
|---|---|---|---|---|---|---|---|---|---|
| | | mRNA-1647 | | | | mRNA-1647 | | | |
| | Placebo (N=22) | 50 µg (N=14) | 100 µg (N=24) | 150 µg (N=18) | Total (N=56) | 50 µg (N=11) | 100 µg (N=12) | 150 µg (N=9) | Total (N=32) |
| *n* | 16 | 7 | 11 | 14 | 32 | 9 | 7 | 8 | 24 |
| GMT (95% CI) | 8.0 NE, NE | 5214.6 (1824.6-14903.1) | 6047.5 (3226.4-11335.1) | 6602.5 (4166.5-10462.7) | 6083.9 (4401.0-8410.3) | 14505.1 (11095.7-18962.2) | 19161.8 (13127.0-27970.8) | 12227.9 (9990.1-14967.1) | 14861.6 (12712.6-17373.9) |
| GMFR (95% CI) | 1.00 NE, NE | 651.83 (228.08-1862.89) | 755.94 (403.31-1416.89) | 825.31 (520.81-1307.84) | 760.49 (550.12-1051.29) | 6.42 (1.19- 34.51) | 2.78 (1.25- 6.20) | 1.33 (0.57- 3.11) | 2.98 (1.50- 5.91) |
| **Neutralizing Antibodies Against Fibroblast Infection**[b] | | | | | | | | | |
| **Baseline** | | | | | | | | | |
| *n* | 22 | 14 | 24 | 18 | 56 | 11 | 12 | 9 | 32 |
| GMT (95% CI) | 8.00 NE, NE | 8.00 NE, NE | 8.00 NE, NE | 8.00 NE, NE | 8.00 NE, NE | 1682.8 (269.5-10506.4) | 5538.4 (3113.0-9853.3) | 8120.2 (4006.5-16457.5) | 4095.3 (2115.0-7929.9) |
| **Month 1** | | | | | | | | | |
| *n* | 22 | 14 | 24 | 15 | 53 | 11 | 12 | 8 | 31 |
| GMT (95% CI) | 8.00 NE, NE | 54.8 (18.6-162.1) | 120.7 (54.4-267.6) | 149.8 (51.3-437.1) | 104.2 (61.7-176.0) | 3916.1 (750.3-20438.3) | 11094.2 (6956.5-17693.0) | 23421.3 (13588.2-40369.9) | 9297.5 (5017.7-17227.9) |
| GMFR (95% CI) | 1.00 NE, NE | 6.86 (2.32-20.27) | 15.09 (6.80-33.45) | 18.72 (6.42-54.64) | 13.02 (7.71-22.00) | 2.33 (1.25-4.34) | 2.00 (1.39-2.88) | 2.47 (1.44-4.23) | 2.23 (1.72-2.90) |
| **Month 2** | | | | | | | | | |
| *n* | 22 | 14 | 24 | 18 | 56 | 11 | 12 | 9 | 32 |
| GMT (95% CI) | 8.00 NE, NE | 47.6 (17.7-127.6) | 79.3 (30.9-203.5) | 94.9 (49.2-182.8) | 73.9 (45.2-121.0) | 3557.5 (658.7-19213.2) | 10935.1 (6330.3-18889.8) | 15301.0 (9705.0-24123.8) | 8169.9 (4479.3-14901.3) |
| GMFR (95% CI) | 1.00 NE, NE | 5.95 (2.22-15.95) | 9.91 (3.86-25.44) | 11.86 (6.15-22.85) | 9.24 (5.65-15.12) | 2.11 (1.23-3.64) | 1.97 (1.38-2.83) | 1.88 (1.09-3.26) | 1.99 (1.56-2.55) |

| Timepoint | CMV-Seronegative | | | | | CMV-Seropositive | | | |
|---|---|---|---|---|---|---|---|---|---|
| | | mRNA-1647 | | | | mRNA-1647 | | | |
| | Placebo (N=22) | 50 µg (N=14) | 100 µg (N=24) | 150 µg (N=18) | Total (N=56) | 50 µg (N=11) | 100 µg (N=12) | 150 µg (N=9) | Total (N=32) |
| **Month 3** | | | | | | | | | |
| *n* | 21 | 9 | 24 | 18 | 51 | 11 | 12 | 9 | 32 |
| GMT (95% CI) | 8.00 NE, NE | 6154.4 (3883.8-9752.3) | 3940.9 (2536.6-6122.6) | 5239.0 (3848.1-7132.6) | 4714.1 (3712.5-5986.0) | 9484.3 (5365.3-16765.6) | 10374.4 (7117.5-15121.5) | 14816.8 (9365.2-23441.7) | 11120.0 (8654.4-14288.1) |
| GMFR (95% CI) | 1.00 NE, NE | 769.30 (485.48-1219.04) | 492.61 (317.08-765.33) | 654.87 (481.01-891.58) | 589.26 (464.06-748.25) | 5.64 (1.19-26.68) | 1.87 (1.19-2.96) | 1.82 (1.10-3.01) | 2.72 (1.57-4.70) |
| **Month 6** | | | | | | | | | |
| *n* | 22 | 14 | 24 | 18 | 56 | 11 | 12 | 9 | 32 |
| GMT (95% CI) | 8.00 NE, NE | 445.5 (207.5-956.5) | 559.5 (275.5-1136.3) | 667.2 (382.4-1164.1) | 559.3 (382.9-816.9) | 7018.9 (3015.7-16336.1) | 10074.6 (6972.3-14557.3) | 16238.0 (11302.2-23329.3) | 10176.0 (7372.7-14045.3) |
| GMFR (95% CI) | 1.00 NE, NE | 55.68 (25.93-119.56) | 69.94 (34.44-142.04) | 83.40 (47.80-145.51) | 69.91 (47.86-102.12) | 4.17 (0.83-20.86) | 1.82 (1.19-2.78) | 2.00 (1.25-3.20) | 2.48 (1.45-4.27) |
| **Month 7** | | | | | | | | | |
| *n* | 22 | 13 | 23 | 18 | 54 | 11 | 12 | 9 | 32 |
| GMT (95% CI) | 8.00 NE, NE | 2594.8 (1429.5-4709.9) | 4085.9 (2830.4-5898.3) | 4066.7 (2858.5-5785.6) | 3657.1 (2901.6-4609.3) | 8263.8 (4416.6-15462.3) | 9575.9 (6366.0-14404.2) | 15429.4 (9711.6-24513.4) | 10409.9 (7896.9-13722.6) |
| GMFR (95% CI) | 1.00 NE, NE | 324.35 (178.69-588.73) | 510.74 (353.80-737.28) | 508.34 (357.31-723.20) | 457.14 (362.70-576.16) | 4.91 (0.94-25.65) | 1.73 (1.02-2.94) | 1.90 (1.09-3.33) | 2.54 (1.43-4.53) |
| **Month 12** | | | | | | | | | |
| *n* | 20 | 14 | 23 | 18 | 55 | 11 | 12 | 9 | 32 |
| GMT | 8.00 | 1402.8 | 1073.7 | 1020.3 | 1130.3 | 7798.9 | 8722.5 | 11974.3 | 9175.6 |

| | CMV-Seronegative | | | | | CMV-Seropositive | | | |
|---|---|---|---|---|---|---|---|---|---|
| | | mRNA-1647 | | | | mRNA-1647 | | | |
| Timepoint | Placebo (N=22) | 50 µg (N=14) | 100 µg (N=24) | 150 µg (N=18) | Total (N=56) | 50 µg (N=11) | 100 µg (N=12) | 150 µg (N=9) | Total (N=32) |
| (95% CI) | NE, NE | (678.0-2902.6) | (644.9-1787.6) | (679.7-1531.6) | (843.2-1515.1) | (4196.4-14494.0) | (5753.1-13224.6) | (7337.9-19540.4) | (7001.7-12024.5) |
| GMFR (95% CI) | 1.00 NE, NE | 175.35 (84.74-362.82) | 134.22 (80.62-223.45) | 127.54 (84.97-191.45) | 141.29 (105.40-189.39) | 4.63 (0.96-22.31) | 1.57 (1.16-2.13) | 1.47 (0.64-3.41) | 2.24 (1.28-3.93) |
| **Month 18** | | | | | | | | | |
| *n* | 21 | 14 | 24 | 18 | 56 | 11 | 12 | 9 | 32 |
| GMT (95% CI) | 8.6 (7.4-9.9) | 868.5 (458.9-1643.9) | 553.4 (299.0, 1024.3) | 716.7 (463.9, 1107.2) | 673.1 (487.6, 929.1) | 6557.5 (3162.2, 13598.5) | 7825.9 (5078.0, 12060.8) | 10211.3 (7006.0, 14883.2) | 7936.6 (5950.6, 10585.4) |
| GMFR (95% CI) | 1.07 (0.93-1.23) | 108.56 (57.36-205.49) | 69.17 (37.37, 128.04) | 89.58 (57.98, 138.41) | 84.13 (60.95, 116.13) | 3.90 (0.84, 18.12) | 1.41 (1.08, 1.86) | 1.26 (0.71, 2.21) | 1.94 (1.14, 3.29) |
| **Month 24** | | | | | | | | | |
| *n* | 20 | 13 | 23 | 18 | 54 | 11 | 12 | 9 | 32 |
| GMT (95% CI) | 8.00 NE, NE | 690.8 (239.1-1995.7) | 542.3 (260.9-1127.0) | 458.4 (313.0-671.4) | 543.5 (366.0-807.1) | 5987.1 (2975.6-12046.3) | 7293.5 (4223.3, 12595.7) | 9923.5 (6635.7, 14840.5) | 7431.6 (5468.9, 10098.7) |
| GMFR (95% CI) | 1.00 NE, NE | 86.35 (29.89, 249.46) | 67.78 (32.61, 140.88) | 57.30 (39.12, 83.92) | 67.94 (45.75, 100.89) | 3.56 (0.75, 16.97) | 1.32 (0.93, 1.87) | 1.22 (0.63, 2.36) | 1.81 (1.05, 3.13) |
| **Month 30** | | | | | | | | | |
| *n* | 21 | 11 | 21 | 17 | 49 | 11 | 12 | 8 | 31 |
| GMT (95% CI) | 10.9 (5.7-20.7) | 383.2 (182.7-803.8) | 733.5 (384.9-1397.5) | 475.6 (246.5-917.6) | 545.5 (374.8-794.0) | 5989.7 (2383.4-15053.0) | 8177.9 (5459.0-12250.9) | 10286.0 (8310.5-12731.3) | 7768.9 (5553.5-10868.1) |

| Timepoint | CMV-Seronegative | | | | | CMV-Seropositive | | | |
|---|---|---|---|---|---|---|---|---|---|
| | | mRNA-1647 | | | | mRNA-1647 | | | |
| | Placebo (N=22) | 50 µg (N=14) | 100 µg (N=24) | 150 µg (N=18) | Total (N=56) | 50 µg (N=11) | 100 µg (N=12) | 150 µg (N=9) | Total (N=32) |
| GMFR (95% CI) | 1.36 (0.72-2.58) | 47.90 (22.84-100.47) | 91.68 (48.12-174.69) | 59.45 (30.81-114.70) | 68.19 (46.85-99.25) | 3.56 (0.82-15.40) | 1.48 (1.08-2.01) | 1.07 (0.64-1.78) | 1.86 (1.10-3.12) |
| **Month 36** | | | | | | | | | |
| *n* | 21 | 11 | 22 | 16 | 49 | 10 | 11 | 8 | 29 |
| GMT (95% CI) | 8.0 NE, NE | 466.2 (157.1-1382.8) | 536.2 (263.6-1090.7) | 358.3 (244.7-524.5) | 455.5 (307.9-673.9) | 3648.5 (1359.5-9791.3) | 5853.0 (3249.1-10543.9) | 10082.8 (6171.9-16471.8) | 5777.7 (3851.6-8667.0) |
| GMFR (95% CI) | 1.00 NE, NE | 58.27 (19.64-172.85) | 67.03 (32.95-136.34) | 44.78 (30.59-65.56) | 56.94 (38.49-84.24) | 2.52 (0.42-15.19) | 1.11 (0.86-1.44) | 1.05 (0.48-2.28) | 1.45 (0.80-2.63) |
| Month 42 | | | | | | | | | |
| *n* | 18 | 8 | 16 | 13 | 37 | 9 | 10 | 8 | 27 |
| GMT (95% CI) | 8.8 (7.2- 10.7) | 151.3 (46.5- 492.2) | 311.9 (145.2- 670.2) | 202.4 (150.7- 271.8) | 229.1 (154.2- 340.5) | 2225.2 (728.4-6798.0) | 6173.4 (2889.1-13191.1) | 5298.4 (2641.5-10627.6) | 4198.9 (2612.3-6749.3) |
| GMFR (95% CI) | 1.10 (0.90- 1.34) | 18.91 (5.81- 61.52) | 38.99 (18.15- 83.78) | 25.30 (18.84- 33.97) | 28.64 (19.28- 42.56) | 0.86 (0.34- 2.21) | 1.16 (0.78- 1.73) | 0.55 (0.31- 0.98) | 0.84 (0.59- 1.20) |
| Month 48 | | | | | | | | | |
| *n* | 17 | 9 | 17 | 14 | 40 | 9 | 10 | 8 | 27 |
| GMT (95% CI) | 8.0 NE, NE | 192.9 (59.0- 630.4) | 181.7 (73.2- 451.2) | 159.8 (107.3- 238.2) | 176.1 (112.8- 274.8) | 5061.6 (2415.3-10607.4) | 4076.0 (1942.4-8553.0) | 5755.9 (3474.7-9534.8) | 4852.8 (3424.9-6876.1) |
| GMFR (95% CI) | 1.00 NE, NE | 24.11 (7.38- 78.80) | 22.71 (9.15- 56.40) | 19.98 (13.41- 29.78) | 22.01 (14.10- 34.34) | 1.96 (0.28- 13.76) | 0.77 (0.44- 1.33) | 0.60 (0.39- 0.92) | 0.98 (0.52- 1.83) |
| Month 54 | | | | | | | | | |
| *n* | 16 | 7 | 11 | 14 | 32 | 9 | 7 | 8 | 24 |

| | CMV-Seronegative | | | | | CMV-Seropositive | | | |
|---|---|---|---|---|---|---|---|---|---|
| | | mRNA-1647 | | | | mRNA-1647 | | | |
| Timepoint | Placebo (N=22) | 50 µg (N=14) | 100 µg (N=24) | 150 µg (N=18) | Total (N=56) | 50 µg (N=11) | 100 µg (N=12) | 150 µg (N=9) | Total (N=32) |
| GMT (95% CI) | 8.0 NE, NE | 135.0 (33.8- 538.2) | 144.0 (55.7- 372.7) | 179.1 (133.0- 241.2) | 156.2 (105.1- 232.1) | 5457.9 (2243.5-13278.1) | 4358.0 (1542.3-12314.0) | 8457.4 (6443.8-11100.3) | 5914.6 (3960.6-8832.6) |
| GMFR (95% CI) | 1.00 NE, NE | 16.87 (4.23- 67.27) | 18.00 (6.96- 46.59) | 22.39 (16.62- 30.15) | 19.53 (13.14- 29.01) | 2.12 (0.37- 12.08) | 0.83 (0.46- 1.48) | 0.88 (0.49- 1.57) | 1.20 (0.64- 2.26) |
| **Anti-gB Binding Antibodies[c]** | | | | | | | | | |
| **Baseline** | | | | | | | | | |
| *n* | 22 | 14 | 24 | 18 | 56 | 11 | 12 | 9 | 32 |
| GMT (95% CI) | 17.50 NE, NE | 17.50 NE, NE | 17.50 NE, NE | 17.50 NE, NE | 17.50 NE, NE | 866.59 (212.35-3536.58) | 2768.81 (1458.77-5255.32) | 4243.22 (2332.50-7719.13) | 2094.22 (1200.47-3653.37) |
| **Month 1** | | | | | | | | | |
| *n* | 22 | 14 | 24 | 15 | 53 | 11 | 12 | 8 | 31 |
| GMT (95% CI) | 17.50 NE, NE | 18.56 (16.34-21.09) | 23.78 (17.30-32.70) | 18.66 (16.26-21.43) | 20.80 (17.90-24.16) | 1546.89 (326.95-7318.81) | 5356.55 (3090.72-9283.47) | 10420.97 (5961.99-18214.83) | 4093.20 (2222.53-7538.38) |
| GMFR (95% CI) | 1.00 NE, NE | 1.061 (0.934-1.205) | 1.359 (0.988-1.869) | 1.067 (0.929-1.225) | 1.189 (1.023-1.381) | 1.785 (1.254-2.541) | 1.935 (1.393-2.687) | 2.283 (1.466-3.556) | 1.962 (1.625-2.370) |
| **Month 2** | | | | | | | | | |
| *n* | 22 | 14 | 24 | 18 | 56 | 11 | 12 | 9 | 32 |
| GMT (95% CI) | 22.33 (13.45-37.07) | 17.50 NE, NE | 27.20 (18.76-39.44) | 17.50 NE, NE | 21.14 (17.95-24.89) | 1422.24 (307.84-6570.92) | 4607.50 (2575.13-8243.89) | 7971.93 (4536.64-14008.53) | 3588.80 (2001.41-6435.21) |
| GMFR (95% CI) | 1.276 (0.769-2.118) | 1.000 NE, NE | 1.554 (1.072-2.254) | 1.000 NE, NE | 1.208 (1.026-1.422) | 1.641 (1.160-2.321) | 1.664 (1.229-2.254) | 1.879 (1.284-2.749) | 1.714 (1.440-2.040) |
| **Month 3** | | | | | | | | | |

| Timepoint | CMV-Seronegative<br>Placebo (N=22) | mRNA-1647<br>50 µg (N=14) | 100 µg (N=24) | 150 µg (N=18) | Total (N=56) | CMV-Seropositive<br>mRNA-1647<br>50 µg (N=11) | 100 µg (N=12) | 150 µg (N=9) | Total (N=32) |
|---|---|---|---|---|---|---|---|---|---|
| *n* | 21 | 9 | 24 | 18 | 51 | 11 | 12 | 9 | 32 |
| GMT (95% CI) | 17.50 NE, NE | 528.05 (290.42-960.12) | 456.68 (266.81-781.68) | 341.36 (228.96-508.94) | 422.80 (315.23-567.06) | 3199.09 (1671.16-6124.00) | 5383.67 (3199.96-9057.56) | 9080.61 (5063.40-16285.00) | 5214.68 (3730.73-7288.88) |
| GMFR (95% CI) | 1.000 NE, NE | 30.174 (16.595-54.864) | 26.096 (15.246-44.668) | 19.507 (13.084-29.082) | 24.160 (18.013-32.404) | 3.692 (1.401-9.726) | 1.944 (1.345-2.810) | 2.140 (1.718-2.665) | 2.490 (1.770-3.503) |
| **Month 6** | | | | | | | | | |
| *n* | 22 | 14 | 24 | 18 | 56 | 11 | 12 | 9 | 32 |
| GMT (95% CI) | 17.50 NE, NE | 114.36 (63.80-205.00) | 120.98 (67.84-215.73) | 121.21 (74.11-198.25) | 119.36 (87.65-162.54) | 3411.67 (1390.97-8367.88) | 5216.46 (3034.27-8968.05) | 9125.32 (5292.87-15732.74) | 5275.86 (3603.79-7723.72) |
| GMFR (95% CI) | 1.000 NE, NE | 6.535 (3.645-11.714) | 6.913 (3.877-12.327) | 6.926 (4.235-11.328) | 6.821 (5.009-9.288) | 3.937 (1.355-11.442) | 1.884 (1.289-2.754) | 2.151 (1.685-2.744) | 2.519 (1.733-3.662) |
| **Month 7** | | | | | | | | | |
| *n* | 22 | 13 | 23 | 18 | 54 | 11 | 12 | 9 | 32 |
| GMT (95% CI) | 17.50 NE, NE | 1180.15 (724.88-1921.35) | 1143.56 (743.24-1759.51) | 995.57 (696.92-1422.18) | 1100.24 (871.43-1389.13) | 4371.84 (2516.75-7594.30) | 5397.83 (3228.49-9024.82) | 10641.93 (5972.41-18962.28) | 6076.59 (4464.68-8270.46) |
| GMFR (95% CI) | 1.000 NE, NE | 67.437 (41.422-109.791) | 65.346 (42.471-100.543) | 56.889 (39.824-81.267) | 62.871 (49.796-79.379) | 5.045 (1.584-16.071) | 1.950 (1.312-2.898) | 2.508 (1.817-3.462) | 2.902 (1.918-4.390) |
| **Month 12** | | | | | | | | | |
| *n* | 20 | 14 | 23 | 18 | 55 | 11 | 12 | 9 | 32 |
| GMT (95% CI) | 17.50 NE, NE | 361.62 | 354.87 | 246.81 | 316.62 | 3873.36 | 5029.03 | 9281.38 | 5462.01 |

|  | CMV-Seronegative |  |  |  |  | CMV-Seropositive |  |  |  |
|---|---|---|---|---|---|---|---|---|---|
|  |  | mRNA-1647 |  |  |  | mRNA-1647 |  |  |  |
| Timepoint | Placebo (N=22) | 50 µg (N=14) | 100 µg (N=24) | 150 µg (N=18) | Total (N=56) | 50 µg (N=11) | 100 µg (N=12) | 150 µg (N=9) | Total (N=32) |
|  |  | (211.10-619.44) | (203.07-620.15) | (174.41-349.26) | (239.31-418.91) | (1666.20-9004.23) | (2905.69-8703.99) | (5492.86-15682.89) | (3795.39-7860.48) |
| GMFR (95% CI) | 1.000 NE, NE | 20.664 (12.063-35.397) | 20.278 (11.604-35.437) | 14.103 (9.966-19.958) | 18.093 (13.675-23.937) | 4.470 (1.375-14.534) | 1.816 (1.325-2.489) | 2.187 (1.709-2.799) | 2.608 (1.737-3.916) |
| **Month 18** |  |  |  |  |  |  |  |  |  |
| *n* | 21 | 14 | 24 | 18 | 56 | 11 | 12 | 9 | 32 |
| GMT (95% CI) | 17.50 NE, NE | 217.72 (124.07-382.07) | 222.62 (110.96-446.62) | 151.47 (108.19-212.05) | 195.61 (140.39-272.55) | 2874.50 (1175.21-7030.88) | 4123.45 (2268.26-7495.98) | 7735.42 (4905.22-12198.58) | 4347.50 (2961.10-6383.02) |
| GMFR (95% CI) | 1.000 NE, NE | 12.441 (7.089-21.833) | 12.721 (6.341-25.521) | 8.655 (6.182-12.117) | 11.178 (8.022-15.574) | 3.317 (1.134-9.702) | 1.489 (1.066-2.081) | 1.823 (1.435-2.316) | 2.076 (1.429-3.015) |
| **Month 24** |  |  |  |  |  |  |  |  |  |
| *n* | 19 | 13 | 23 | 17 | 53 | 11 | 12 | 9 | 32 |
| GMT (95% CI) | 17.50 NE, NE | 224.82 (76.95-656.86) | 157.21 (81.49-303.28) | 130.19 (85.55-198.11) | 161.55 (109.92-237.43) | 3268.23 (1097.46-9732.74) | 4471.18 (2399.42-8331.79) | 8027.57 (4658.63-13832.78) | 4732.79 (3065.10-7307.86) |
| GMFR (95% CI) | 1.000 NE, NE | 12.847 (4.397-37.535) | 8.983 (4.656-17.330) | 7.439 (4.889-11.321) | 9.231 (6.281-13.567) | 3.771 (1.259-11.300) | 1.615 (1.170-2.230) | 1.892 (1.512-2.368) | 2.260 (1.543-3.310) |
| **Month 30** |  |  |  |  |  |  |  |  |  |
| *n* | 21 | 11 | 21 | 17 | 49 | 11 | 12 | 8 | 31 |
| GMT (95% CI) | 22.38 (13.40-37.38) | 143.05 (72.82-281.01) | 190.56 (95.67-379.56) | 116.80 (63.14-216.09) | 150.77 (103.79-219.03) | 2755.72 (1048.38-7243.54) | 3549.90 (1932.92-6519.57) | 7501.18 (4156.36-13537.72) | 3935.86 (2589.16-5983.01) |
| GMFR | 1.279 | 8.174 | 10.889 | 6.675 | 8.615 | 3.180 | 1.282 | 1.530 | 1.852 |

| | CMV-Seronegative | | | | | CMV-Seropositive | | | |
|---|---|---|---|---|---|---|---|---|---|
| | | mRNA-1647 | | | | mRNA-1647 | | | |
| Timepoint | Placebo (N=22) | 50 µg (N=14) | 100 µg (N=24) | 150 µg (N=18) | Total (N=56) | 50 µg (N=11) | 100 µg (N=12) | 150 µg (N=9) | Total (N=32) |
| (95% CI) | (0.766-2.136) | (4.161-16.058) | (5.467-21.689) | (3.608-12.348) | (5.931-12.516) | (1.063-9.517) | (0.973-1.690) | (1.243-1.884) | (1.252-2.742) |
| **Month 36** | | | | | | | | | |
| *n* | 19 | 11 | 22 | 16 | 49 | 10 | 11 | 8 | 29 |
| GMT (95% CI) | 17.50 NE, NE | 129.33 (64.71-258.49) | 156.50 (82.27-297.68) | 96.07 (62.52-147.62) | 127.86 (91.12-179.40) | 2380.33 (861.74-6575.05) | 4233.31 (2075.05-8636.38) | 7195.53 (4004.42-12929.63) | 4018.02 (2574.47-6271.00) |
| GMFR (95% CI) | 1.000 NE, NE | 7.390 (3.698-14.771) | 8.943 (4.701-17.010) | 5.490 (3.573-8.436) | 7.306 (5.207-10.251) | 3.520 (1.083-11.442) | 1.538 (1.158-2.044) | 1.468 (1.179-1.827) | 2.020 (1.350-3.023) |
| Month 42 | | | | | | | | | |
| *n* | 18 | 9 | 18 | 13 | 40 | 9 | 10 | 8 | 27 |
| GMT (95% CI) | 30.97 (14.44- 66.40) | 66.60 (32.65-135.82) | 106.29 (51.72-218.41) | 68.56 (48.72- 96.48) | 82.97 (58.10- 118.47) | 1674.77 (681.90-4113.30) | 3404.12 (1465.76-7905.86) | 4461.99 (2465.09-8076.53) | 2911.68 (1885.14-4497.22) |
| GMFR (95% CI) | 1.770 (0.825- 3.794) | 3.805 (1.866- 7.761) | 6.074 (2.956-12.480) | 3.917 (2.784- 5.513) | 4.741 (3.320- 6.770) | 1.650 (1.018- 2.675) | 1.257 (0.927- 1.705) | 0.910 (0.730- 1.135) | 1.251 (1.022- 1.531) |
| Month 48 | | | | | | | | | |
| *n* | 17 | 9 | 17 | 14 | 40 | 9 | 10 | 8 | 27 |
| GMT (95% CI) | 17.50 NE, NE | 66.12 (35.03-124.81) | 106.34 (44.59-253.61) | 63.31 (40.87- 98.06) | 79.69 (53.40- 118.93) | 2534.83 (1404.97-4573.32) | 3120.23 (1340.95-7260.40) | 2414.24 (424.33-13736.03) | 2698.35 (1577.47-4615.67) |
| GMFR (95% CI) | 1.000 NE, NE | 3.778 (2.001- 7.132) | 6.076 (2.548-14.492) | 3.618 (2.335- 5.604) | 4.554 (3.051- 6.796) | 2.498 (0.614-10.159) | 1.152 (0.835- 1.590) | 0.493 (0.078- 3.129) | 1.159 (0.595- 2.258) |
| Month 54 | | | | | | | | | |

| | CMV-Seronegative | | | | | CMV-Seropositive | | | |
|---|---|---|---|---|---|---|---|---|---|
| | | mRNA-1647 | | | | mRNA-1647 | | | |
| Timepoint | Placebo (N=22) | 50 µg (N=14) | 100 µg (N=24) | 150 µg (N=18) | Total (N=56) | 50 µg (N=11) | 100 µg (N=12) | 150 µg (N=9) | Total (N=32) |
| *n* | 16 | 7 | 11 | 14 | 32 | 9 | 7 | 8 | 24 |
| GMT (95% CI) | 18.51 (16.42- 20.87) | 67.69 (33.20- 138.01) | 94.32 (31.04- 286.66) | 60.79 (41.63- 88.76) | 72.38 (48.62- 107.75) | 2394.63 (1266.68- 4526.99) | 3692.23 (1069.77- 12743.46) | 5360.13 (3201.87- 8973.19) | 3554.12 (2358.80- 5355.18) |
| GMFR (95% CI) | 1.058 (0.938- 1.192) | 3.868 (1.897- 7.886) | 5.390 (1.774- 16.380) | 3.474 (2.379- 5.072) | 4.136 (2.778- 6.157) | 2.360 (0.602- 9.250) | 1.402 (0.844- 2.328) | 1.094 (0.827- 1.447) | 1.569 (0.965- 2.551) |
| **Anti-Pentamer Binding Antibodies[d]** | | | | | | | | | |
| **Baseline** | | | | | | | | | |
| *n* | 22 | 14 | 24 | 18 | 56 | 11 | 12 | 9 | 32 |
| GMT (95% CI) | 24.00 NE, NE | 24.00 NE, NE | 24.00 NE, NE | 24.00 NE, NE | 24.00 NE, NE | 206.5 (87.2-489.3) | 410.7 (228.2-739.1) | 625.9 (266.0- 1472.6) | 365.0 (239.7-555.8) |
| **Month 1** | | | | | | | | | |
| *n* | 22 | 14 | 24 | 15 | 53 | 11 | 12 | 8 | 31 |
| GMT (95% CI) | 24.00 NE, NE | 175.9 (67.4-459.0) | 97.9 (53.2-180.2) | 469.9 (274.3-805.0) | 178.2 (117.3-270.7) | 1483.7 (514.1- 4282.6) | 4688.0 (2218.8- 9905.2) | 5895.4 (3045.0- 11414.0) | 3306.6 (2005.8- 5451.0) |
| GMFR (95% CI) | 1.00 NE, NE | 7.33 (2.81-19.12) | 4.08 (2.22-7.51) | 19.58 (11.43-33.54) | 7.42 (4.89-11.28) | 7.19 (3.87-13.34) | 11.42 (4.88-26.70) | 8.30 (2.87-24.00) | 8.92 (5.82-13.68) |
| **Month 2** | | | | | | | | | |
| *n* | 22 | 14 | 24 | 18 | 56 | 11 | 12 | 9 | 32 |
| GMT (95% CI) | 24.00 NE, NE | 131.5 (58.2-297.4) | 80.5 (48.7-132.9) | 252.4 (157.7-404.0) | 131.4 (93.8-184.0) | 1081.5 (394.7- 2963.3) | 3158.6 (1636.4- 6096.5) | 3696.4 (2200.3- 6209.8) | 2284.0 (1462.2- 3567.6) |
| GMFR (95% CI) | 1.00 NE, NE | 5.48 (2.42-12.39) | 3.35 (2.03-5.54) | 10.52 (6.57-16.83) | 5.48 (3.91-7.67) | 5.24 (3.10-8.86) | 7.69 (3.58-16.52) | 5.91 (2.68-13.02) | 6.26 (4.35-8.99) |

| | CMV-Seronegative | | | | | CMV-Seropositive | | | |
|---|---|---|---|---|---|---|---|---|---|
| | | mRNA-1647 | | | | mRNA-1647 | | | |
| Timepoint | Placebo (N=22) | 50 µg (N=14) | 100 µg (N=24) | 150 µg (N=18) | Total (N=56) | 50 µg (N=11) | 100 µg (N=12) | 150 µg (N=9) | Total (N=32) |
| **Month 3** | | | | | | | | | |
| *n* | 21 | 9 | 24 | 18 | 51 | 11 | 12 | 9 | 32 |
| GMT (95% CI) | 24.00 NE, NE | 8934.8 (5599.7-14256.3) | 3029.8 (1444.2-6356.2) | 5488.0 (3889.6-7743.3) | 4522.3 (3096.8-6603.8) | 3460.4 (2336.0-5126.0) | 4057.3 (2234.7-7366.4) | 4122.6 (2414.8-7038.1) | 3858.6 (2950.2-5046.9) |
| GMFR (95% CI) | 1.00 NE, NE | 372.28 (233.32-594.01) | 126.24 (60.17-264.84) | 228.67 (162.07-322.64) | 188.43 (129.03-275.16) | 16.76 (6.08-46.17) | 9.88 (5.41-18.05) | 6.59 (3.23-13.45) | 10.57 (6.88-16.24) |
| **Month 6** | | | | | | | | | |
| *n* | 22 | 14 | 24 | 18 | 56 | 11 | 12 | 9 | 32 |
| GMT (95% CI) | 24.00 NE, NE | 712.6 (422.3-1202.3) | 473.8 (271.9-825.8) | 653.1 (439.8-969.9) | 581.7 (436.7-774.9) | 1872.5 (938.0-3738.0) | 2953.6 (1683.0-5183.3) | 2490.9 (1726.7-3593.5) | 2407.1 (1773.8-3266.7) |
| GMFR (95% CI) | 1.00 NE, NE | 29.69 (17.60-50.09) | 19.74 (11.33-34.41) | 27.21 (18.32-40.41) | 24.24 (18.20-32.29) | 9.07 (3.22-25.52) | 7.19 (3.82-13.54) | 3.98 (1.86-8.51) | 6.59 (4.25-10.23) |
| **Month 7** | | | | | | | | | |
| *n* | 22 | 13 | 23 | 18 | 54 | 11 | 12 | 9 | 32 |
| GMT (95% CI) | 24.00 NE, NE | 5846.1 (2967.9-11515.6) | 3094.4 (1729.4-5536.6) | 6667.1 (4353.6-10210.0) | 4658.1 (3372.2-6434.4) | 2970.4 (1630.2-5412.7) | 2981.8 (1740.9-5107.5) | 3358.3 (1987.0-5675.9) | 3079.2 (2310.1-4104.2) |
| GMFR (95% CI) | 1.00 NE, NE | 243.59 (123.66-479.82) | 128.93 (72.06-230.69) | 277.80 (181.40-425.42) | 194.09 (140.51-268.10) | 14.38 (4.98-41.59) | 7.26 (4.06-12.99) | 5.37 (2.90-9.93) | 8.44 (5.47-13.00) |
| **Month 12** | | | | | | | | | |
| *n* | 20 | 14 | 23 | 18 | 55 | 11 | 12 | 9 | 32 |
| GMT (95% CI) | 24.00 NE, NE | 1454.7 | 1365.3 | 1314.8 | 1370.5 | 1399.9 | 2158.7 | 2244.3 | 1880.6 |

| Timepoint | CMV-Seronegative | | | | | CMV-Seropositive | | | |
|---|---|---|---|---|---|---|---|---|---|
| | | mRNA-1647 | | | | mRNA-1647 | | | |
| | Placebo (N=22) | 50 µg (N=14) | 100 µg (N=24) | 150 µg (N=18) | Total (N=56) | 50 µg (N=11) | 100 µg (N=12) | 150 µg (N=9) | Total (N=32) |
| | | (779.9-2713.4) | (773.3-2410.4) | (849.3-2035.3) | (1015.6-1849.5) | (785.8-2494.2) | (1153.5-4039.8) | (1661.4-3031.7) | (1399.8-2526.4) |
| GMFR (95% CI) | 1.00 NE, NE | 60.61 (32.50-113.06) | 56.89 (32.22-100.43) | 54.78 (35.39-84.81) | 57.10 (42.32-77.06) | 6.78 (2.65-17.38) | 5.26 (2.42-11.43) | 3.59 (1.70-7.58) | 5.15 (3.33-7.98) |
| **Month 18** | | | | | | | | | |
| *n* | 21 | 14 | 24 | 18 | 56 | 11 | 12 | 9 | 32 |
| GMT (95% CI) | 24.00 NE, NE | 716.2 (403.1-1272.4) | 590.9 (327.1-1067.6) | 661.1 (437.7-998.6) | 642.8 (475.1-869.6) | 850.6 (476.8-1517.6) | 1426.3 (1017.6-1999.3) | 1615.0 (1185.9-2199.5) | 1236.6 (968.4-1579.0) |
| GMFR (95% CI) | 1.00 NE. NE | 29.84 (16.80-53.02) | 24.62 (13.63-44.49) | 27.55 (18.24-41.61) | 26.78 (19.80-36.23) | 4.12 (1.93-8.77) | 3.47 (2.05-5.89) | 2.58 (1.26-5.26) | 3.39 (2.40-4.78) |
| **Month 24** | | | | | | | | | |
| *n* | 20 | 13 | 23 | 17 | 53 | 11 | 12 | 9 | 32 |
| GMT (95% CI) | 24.00 NE, NE | 633.9 (329.0-1221.6) | 413.8 (224.7-762.3) | 523.6 (366.8-747.4) | 495.5 (362.1-678.0) | 657.3 (318.1-1358.2) | 1257.9 (716.1-2209.4) | 1480.7 (956.2-2293.1) | 1053.6 (753.6-1473.0) |
| GMFR (95% CI) | 1.00 NE, NE | 26.41 (13.71-50.90) | 17.24 (9.36-31.76) | 21.82 (15.28-31.14) | 20.65 (15.09-28.25) | 3.18 (1.55-6.56) | 3.06 (1.63-5.75) | 2.37 (1.36-4.12) | 2.89 (2.06-4.04) |
| **Month 30** | | | | | | | | | |
| *n* | 21 | 11 | 21 | 17 | 49 | 11 | 12 | 8 | 31 |
| GMT (95% CI) | 26.8 (21.3-33.7) | 388.7 (210.5-717.8) | 522.3 (311.9-874.7) | 377.2 (258.5-550.4) | 436.6 (331.8-574.5) | 621.5 (334.8-1153.9) | 977.8 (534.6-1788.3) | 1299.3 (653.3-2584.2) | 896.0 (637.6-1259.1) |
| GMFR (95% CI) | 1.12 (0.89-1.41) | 16.20 (8.77-29.91) | 21.76 (13.00-36.44) | 15.72 (10.77-22.93) | 18.19 (13.83-23.94) | 3.01 (1.51-5.99) | 2.38 (1.23-4.60) | 1.61 (1.10-2.34) | 2.34 (1.67-3.28) |
| **Month 36** | | | | | | | | | |

| | CMV-Seronegative | | | | | CMV-Seropositive | | | |
|---|---|---|---|---|---|---|---|---|---|
| | | mRNA-1647 | | | | mRNA-1647 | | | |
| Timepoint | Placebo<br>(N=22) | 50 µg<br>(N=14) | 100 µg<br>(N=24) | 150 µg<br>(N=18) | Total<br>(N=56) | 50 µg<br>(N=11) | 100 µg<br>(N=12) | 150 µg<br>(N=9) | Total<br>(N=32) |
| *n* | 21 | 11 | 22 | 16 | 49 | 10 | 11 | 8 | 29 |
| GMT<br>(95% CI) | 24.00<br>NE, NE | 357.8<br>(160.7-796.4) | 361.3<br>(214.7-608.2) | 309.2<br>(210.2-455.1) | 342.7<br>(254.6-461.1) | 432.9<br>(249.5-751.1) | 1002.8<br>(505.9-1987.7) | 1141.5<br>(745.1-1748.7) | 777.9<br>(551.6-1096.9) |
| GMFR<br>(95% CI) | 1.00<br>NE, NE | 14.91<br>(6.70-33.18) | 15.06<br>(8.94-25.34) | 12.89<br>(8.76-18.96) | 14.28<br>(10.61-19.21) | 2.51<br>(1.21-5.19) | 2.47<br>(1.19-5.09) | 1.41<br>(0.89-2.24) | 2.12<br>(1.48-3.04) |
| Month 42 | | | | | | | | | |
| *n* | 18 | 9 | 18 | 13 | 40 | 9 | 10 | 8 | 27 |
| GMT<br>(95% CI) | 25.0<br>(22.9- 27.3) | 192.5<br>(83.3- 445.1) | 135.0<br>(77.0- 236.7) | 127.8<br>(59.4- 275.2) | 143.7<br>(99.5- 207.5) | 506.2<br>(268.0- 955.9) | 774.5<br>(423.1-1417.8) | 681.2<br>(454.6-1020.6) | 647.0<br>(481.9- 868.8) |
| GMFR<br>(95% CI) | 1.04<br>(0.95- 1.14) | 8.02<br>(3.47- 18.55) | 5.62<br>(3.21- 9.86) | 5.33<br>(2.47- 11.47) | 5.99<br>(4.14- 8.64) | 2.35<br>(1.31- 4.23) | 2.08<br>(0.96- 4.52) | 0.84<br>(0.51- 1.38) | 1.66<br>(1.14- 2.41) |
| Month 48 | | | | | | | | | |
| *n* | 17 | 9 | 17 | 14 | 40 | 9 | 10 | 8 | 27 |
| GMT<br>(95% CI) | 24.0<br>NE, NE | 165.4<br>(70.0- 391.1) | 143.0<br>(76.9- 266.1) | 160.6<br>(91.9- 280.6) | 153.9<br>(108.9- 217.5) | 449.8<br>(290.6- 696.0) | 675.4<br>(347.6-1312.5) | 814.4<br>(504.0-1315.9) | 623.4<br>(465.2- 835.5) |
| GMFR<br>(95% CI) | 1.00<br>NE, NE | 6.89<br>(2.91- 16.30) | 5.96<br>(3.20- 11.09) | 6.69<br>(3.83- 11.69) | 6.41<br>(4.54- 9.06) | 2.09<br>(0.94- 4.62) | 1.82<br>(0.85- 3.88) | 1.01<br>(0.67- 1.51) | 1.60<br>(1.10- 2.32) |
| Month 54 | | | | | | | | | |
| *n* | 16 | 7 | 11 | 14 | 32 | 9 | 7 | 8 | 24 |
| GMT<br>(95% CI) | 24.0<br>NE, NE | 110.0<br>(43.8- 276.1) | 104.4<br>(55.6- 196.1) | 148.4<br>(90.3- 243.8) | 123.2<br>(89.0- 170.5) | 353.7<br>(191.9- 652.2) | 960.1<br>(584.3-1577.7) | 665.0<br>(430.6-1027.0) | 584.2<br>(426.2- 800.6) |
| GMFR<br>(95% CI) | 1.00<br>NE, NE | 4.58<br>(1.83- 11.50) | 4.35<br>(2.32- 8.17) | 6.18<br>(3.76- 10.16) | 5.13<br>(3.71- 7.10) | 1.64<br>(0.62- 4.36) | 2.36<br>(1.25- 4.46) | 0.82<br>(0.57- 1.17) | 1.45<br>(0.96- 2.18) |

Abbreviations: CI, confidence interval; GMT, geometric mean titer; GMFR, geometric mean fold-rise; LLOQ, lower limit of quantification; ULOQ, upper limit of quantification

[a]Neutralizing antibodies epithelial cell: LLOQ = 16; ULOQ=33,554,432.

[b]Neutralizing antibodies against fibroblast: LLOQ=16; ULOQ=16,777,216.

[c]Anti-gB antibodies in fold dilution: LLOQ=35; ULOQ: 991,287.

[d]Anti-Pentamer antibodies in fold dilution: LLOQ=48; ULOQ=117,612.

N is the number of participants in the primary extension phase per-protocol set for antibody-mediated immunogenicity which consisted of participants from the respective analysis population from the main phase 2 study who were enrolled in the primary extension phase.

*n* is the number of participants with non-missing data at baseline or at the corresponding timepoint.

95% CIs were calculated based on the t-distribution of the log-transformed values or the difference in the log-transformed values for GMT and GMFR, respectively, then back-transformed to the original scale for presentation.

**Table S6. Incidence Rate of CMV Seroconversion in CMV Seronegative Participants in Primary Extension Phase Primary Extension Phase Per-Protocol Set for CMV Seroconversion**

| | | | mRNA-1647 | | |
|---|---|---|---|---|---|
| Timepoint | Statistic | Placebo (N=23) n (%) | 50 µg (N=15) n (%) | 100 µg (N=24) n (%) | 150 µg (N=18) n (%) |
| Month 24 | Number of Participants with CMV Seroconversion [1] | 0 | 0 | 0 | 0 |
| | Incidence Rate per 1,000 Person-Years (95% CI) [2] | 0 (NE, 523.5) | 0 (NE, 818.1) | 0 (NE, 399.2) | 0 (NE, 547.7) |
| Month 30 | Number of Participants with CMV Seroconversion [1] | 1 | 1 | 0 | 0 |
| | Incidence Rate per 1,000 Person-Years (95% CI) [2] | 56.4 (1.4, 314.1) | 94.0 (2.4, 523.6) | 0 (NE, 182.3) | 0 (NE, 236.4) |
| Month 36 | Number of Participants with CMV Seroconversion [1] | 1 | 1 | 0 | 0 |
| | Incidence Rate per 1,000 Person-Years (95% CI) [2] | 35.1 (0.9, 195.5) | 59.0 (1.5, 329.0) | 0 (NE, 119.0) | 0 (NE, 154.7) |
| Month 42 | Number of Participants with CMV Seroconversion [1] | 1 | 1 | 0 | 0 |
| | Incidence Rate per 1,000 Person-Years (95% CI) [2] | 25.8 (0.7, 143.7) | 43.9 (1.1, 244.5) | 0 (NE, 89.8) | 0 (NE, 118.1) |
| Month 48 | Number of Participants with CMV Seroconversion [1] | 1 | 1 | 0 | 0 |
| | Incidence Rate per 1,000 Person-Years (95% CI) [2] | 20.7 (0.5, 115.1) | 35.6 (0.9, 198.1) | 0 (NE, 73.9) | 0 (NE, 95.5) |
| Month 54 | Number of Participants with CMV Seroconversion [1] | 1 | 1 | 1 | 0 |
| | Incidence Rate per 1,000 Person-Years (95% CI) [2] | 18.1 (0.5, 100.7) | 31.3 (0.8, 174.4) | 18.0 (0.5, 100.2) | 0 (NE, 83.5) |

[1] CMV seroconversion from informed consent form (ICF) signature through the end of the Primary Extension Phase is defined as seroconversion from a negative to a positive result for serum immunoglobulin (Ig)G against at least one of four recombinant CMV antigens not encoded by mRNA-1647 (pp150, pp28, pp52, pp38) as measured by a platform-based automated immunoassay.

[2] Incidence rate is calculated as the number of participants with the event (i.e., first occurrence of CMV seroconversion) divided by the number of participants at risk adjusted for person-years. Person-years is calculated from Study P202-EXT ICF signature until the earliest of first CMV seroconversion event, the end of the time period, phase completion, discontinuation from the phase, death, or data cutoff date. The 95% CI is calculated using the exact method (Poisson distribution) and adjusted for person-years.
